# Supplementary material for: LoCoHD: a metric for comparing local environments of proteins
Source: Nat Commun. 2024 May 13;15:4029. doi: 10.1038/s41467-024-48225-0 (PMC11091161; doi:10.1038/s41467-024-48225-0)
Supplement: Supplementary file 1 — Supplementary Information [file 41467_2024_48225_MOESM1_ESM.pdf]

Supplementary Material

LoCoHD: a Metric for Comparing Local Environments Of Proteins

Zsolt Fazekas[1,2], Dóra K. Menyhárd[1,3], András Perczel[1,3*]

[1]Laboratory of Structural Chemistry and Biology, Institute of Chemistry, ELTE Eötvös Loránd University, Hungary,
[2]ELTE Hevesy György PhD School of Chemistry, ELTE Eötvös Loránd University, Hungary,
[3]MTA-ELTE Protein Modeling Research Group, Eötvös Loránd Research Network (ELKH), ELTE Eötvös Loránd University, Hungary
*E-mail: perczel.andras@ttk.elte.hu

Supplementary Note 1: Overview of Protein Similarity and Dissimilarity Measures

One of the most abundantly used metric is the root mean squared deviation (RMSD)[1] of different atom-groups of proteins, like the Cα, backbone, backbone plus Cβ, or heavy-atom groups. This is a proper metric (i.e. it satisfies the strict definitions of a metric space)[2,3] and is alignment-based, which means that the structures to be compared must be superimposed using the Kabsch algorithm before the final RMSD is calculated. It is evaluated as follows:

$$\mathrm{RMSD}(S_1, S_2) = \sqrt{\frac{1}{|A|}\sum_{a\in A}\|\mathbf{r}_{a1} - \mathbf{r}_{a2}\|^2}$$

where, S_1 and S_2 are the structures for which we want to calculate the RMSD, 'A' is the atom-set on which we want to calculate the RMSD, and $\mathbf{r}_{ai}$ is the position of the atom 'a' in the ith structure. It follows that this is basically the L2-norm of two 3|A| dimensional vectors, representing the A atom-sets of S_1 and S_2. The advantages of this metric are that it is intuitive to formulate, easy to compute, and widely used in a variety of applications. However, one should be careful to claim non-similarity between two protein structures (or folds) based on their RMSDs alone. Two protein structures may appear visually similar but have a large RMSD if, for example, a tail region or a flexible loop adopts different conformations. Large but only local changes can shift the RMSD to higher values. On the other hand, different conformations of a protein consisting of several domains connected by flexible hinge regions can lead to large RMSD values, even if the domain folds are very similar. The size of the proteins being compared also introduces a systematic bias into this metric[4]. Lastly, one cannot easily infer differences regarding the fold-shaping residue-residue interactions from an RMSD calculation.

To overcome some of the disadvantages of RMSD, several new measures and structure comparison algorithms were developed, including DALI[5], PAUL[6], the GDT-score[7], the MaxSub-score[8], the MAMMOTH-score[9], the TM-score and TMalign algorithm[4], or the lDDT score[10], to name a few. These all aim to tackle the problem of protein structure-similarity or -dissimilarity measurement in an automated manner and patching the disadvantages associated with the use of RMSD. Some of these methods primarily aim for structural alignment and not similarity scoring, although all alignment methods try to minimize (or maximize) some sort of target function, the final value of which can be regarded as a dissimilarity (or similarity) score.

The GDT, MaxSub, MAMMOTH, and TM methods all use full- or partial 3D-structure alignment. The GDT (global distance test) method searches for the maximum subset of specific protein atoms (usually Cα atoms) that can be aligned to a target set within a specified maximum deviation threshold. The percentage of the successfully aligned atoms is then reported. The GDT_TS score is the average of four different percentages provided by four different GDT runs with different thresholds. This scoring method is still widely used in the CASP competitions - the biannual, community-wide evaluation of structure prediction methods - and is a gold standard for the testing of other alignment-based scoring functions.

In the MaxSub (maximum substructure) algorithm the same principles govern the fitting; it aims to identify the largest superimposable subset of Cα atoms. Similarly to the GDT's maximum deviation threshold-based algorithm, the MaxSub algorithm searches the largest subset that aligns below a certain maximum deviation threshold, but for which the RMSD is also minimal. It finds this subset using an independently developed heuristic algorithm. Furthermore, the reported score is a variation of the Levitt-Gerstein (LG) score[11], which weights large deviations less strictly than the RMSD metric:

$$\mathrm{LG}(S_1, S_2) = \frac{1}{|I|}\sum_{i\in I}\left(1 + \left(\frac{\|\mathbf{r}_{i1} - \mathbf{r}_{i2}\|}{d}\right)^2\right)^{-1}$$

where I is the collection of residue indices, for which the residues are aligned below the RMSD threshold, $\mathbf{r}_{ij}$ is the position of the Cα atom of the ith residue in the jth structure, and d is the applied threshold distance (originally 3.5 Å). This results in a score between 0 and 1, with more similar structures being closer to 1.

Supplementary Note 1: Overview of Protein Similarity and Dissimilarity Measures (Continuation)

In MAMMOTH (matching molecular models obtained from theory) all residue pairs between the two structures are compared through their neighboring Cα traces. A normalized unit-vector root mean square (URMS) distance matrix is constructed for all heptapeptide pairs between the two structures, then this matrix is converted to a similarity matrix and the Needleman-Wunsch (NW)[12] algorithm is applied to it to obtain a global sequential alignment. The MaxSub algorithm is then used to align the sequentially aligned segments in the 3D space, and the probability (P-value) of obtaining the given proportion of aligned residues is reported as a single score.

To take full advantage of the LG-scoring scheme, Zhang *et al.* developed the TM-score (template modelling score) and TMalign algorithm. In this algorithm the alignment procedure uses the LG-score variant directly instead of the RMSD. Since the GDT_TS and MaxSub scores both have a power-law dependence on protein length (in this case, the length of the smaller protein to be compared), making it difficult to interpret their absolute values, the *d* parameter in the TM-score was designed to be dependent on protein length (*L*), making the final score and protein length essentially uncorrelated.:

$$d(L) = 1.24\ \text{Å}\ \sqrt[3]{L-15} - 1.18\ \text{Å}$$

Here, all residues are aligned, not only a subset of them. Similarly to the GDT_TS score, the TM-score is also regularly used during the CASP competitions.

In parallel to structure alignment-based scoring, scoring methods that use some kind of structure-derived distance-matrix were also developed. The distance-matrix of a protein is the distance-matrix of a specific coordinate-set defined by or within the structure, like the coordinates of the Cα atoms, all atoms, or the coordinates of residue centroids. All distance matrices are invariant to protein structure rotation and translation, in contrast to the explicit atomic (or centroid) coordinates. It is tempting to exploit this invariance, since comparison of protein structures through their distance matrices eliminates the need for a structure alignment. The DALI software is an early adopter of this concept, as it aligns protein segments through their distance-matrix submatrices through Monte Carlo (MC) searching steps. It constructs the Cα distance matrix of the full protein structures, which are then segmented into submatrices, which are then paired based on their so-called elastic similarity scores:

$$\phi(\mathbf{A},\mathbf{B}) = \sum_{\forall ij} \left(\theta - 2\frac{|A_{ij} - B_{ij}|}{A_{ij} + B_{ij}}\right) \exp\left(-\beta\left(A_{ij} + B_{ij}\right)^2\right)$$

where **A** and **B** are the submatrices to be compared, and *Θ* and *β* are adjustable parameters. In the case of $i = j$, the value of *φ* is chosen to be *Θ*. Due to combinatorial explosion, the optimal submatrix-pairing and -threading can only be approximated through heuristics, which are achieved by MC methods.

The PAUL (Protein Structural Alignment Using Lagrangian Relaxation) method, which also performs distance matrix alignment, replaces the MC algorithm of DALI with an iterative double dynamic programming algorithm. PAUL uses a two-stage alignment scheme that is executed alternately until convergence is reached. In the lower level algorithm, the rigid similarity scoring scheme is used (which was also developed for DALI) for NW distance alignment of specific residue-residue pairs (a cell in the upper level matrix). In the upper level a “classic” NW sequential alignment is performed using the scores provided by the lower level distance alignments. When the upper- and lower-level alignments match, PAUL terminates.

Supplementary Note 1: Overview of Protein Similarity and Dissimilarity Measures (Continuation)

In addition to the RMSD, GST_TS, and TM-score scoring schemes, the lDDT score (local distance difference test) is also widely used in CASP evaluations. This similarity score is also distance-matrix based, although its calculation is governed by different principles than those of DALI or PAUL. Since it was specifically designed to compare proteins with the same sequence, but different folds, it relies heavily on the one-to-one correspondence between the two protein's atom sets. The lDDT score is the percentage of preserved atom-atom distances, considering only distances below R_0 (a distance threshold called "inclusion radius"). A distance d is considered preserved if, when compared to its corresponding distance d_0 from the other model, the deviation of d from d_0 is smaller than a tolerance threshold T. The reported final score is the average lDDT computed over four different T values, namely 0.5 Å, 1 Å, 2 Å and 4 Å. The one-to-one correspondence of the two atom sets is not a necessity for the evaluation of lDDT, since distances (i.e. atom-pairs) that are present in the reference structure, but not in the query structure, are automatically considered non-preserved. This means, that proteins showing high structural-, but low sequential similarity will always have a low lDDT score.

Other notable scoring systems include the contact area difference (CAD) score[13], which compares the inter-residue contact areas between the model and target structure, the universal similarity metric (USM)[14], which extracts and compares the Kolmogorov complexity of protein contact maps, variants of the principle component correlation (PCC) techniques[15], assessing similarity through correlating principal components of secondary structure interaction matrices, or SProt[16], which calculates the atomic content spatial alignment of amino acid environments (spheres). Besides these, deep learning based approaches - aiming to predict existing or novel scores - are also starting to emerge, like DeeplyTough[17] for protein pockets, TM-Vec[18] for predicting TM-scores solely from sequences, or EnQA[19] for estimating the accuracy of a model through predicted lDDT scores.

Method name	Alignment applied*	Score type	Theoretical score range	Reference
RMSD	1	dissimilarity	[0, ∞)	1, 2, 3
GDT_TS	1	similarity	[0, 1]	7
MaxSub	1	similarity	[0, 1]	8
MAMMOTH	1	similarity	[0, 1]	9
TM-score	1	similarity	[0, 1]	4
DALI	2	similarity	(-∞, ∞)	5
PAUL	2	similarity	(-∞, ∞)	6
lDDT	3	similarity	[0, 1]	10

Supplementary Table 1 List of protein structure comparison scores. *The applied alignment categories are (1): coordinate alignment, (2): distance matrix alignment, (3): no alignment is performed.

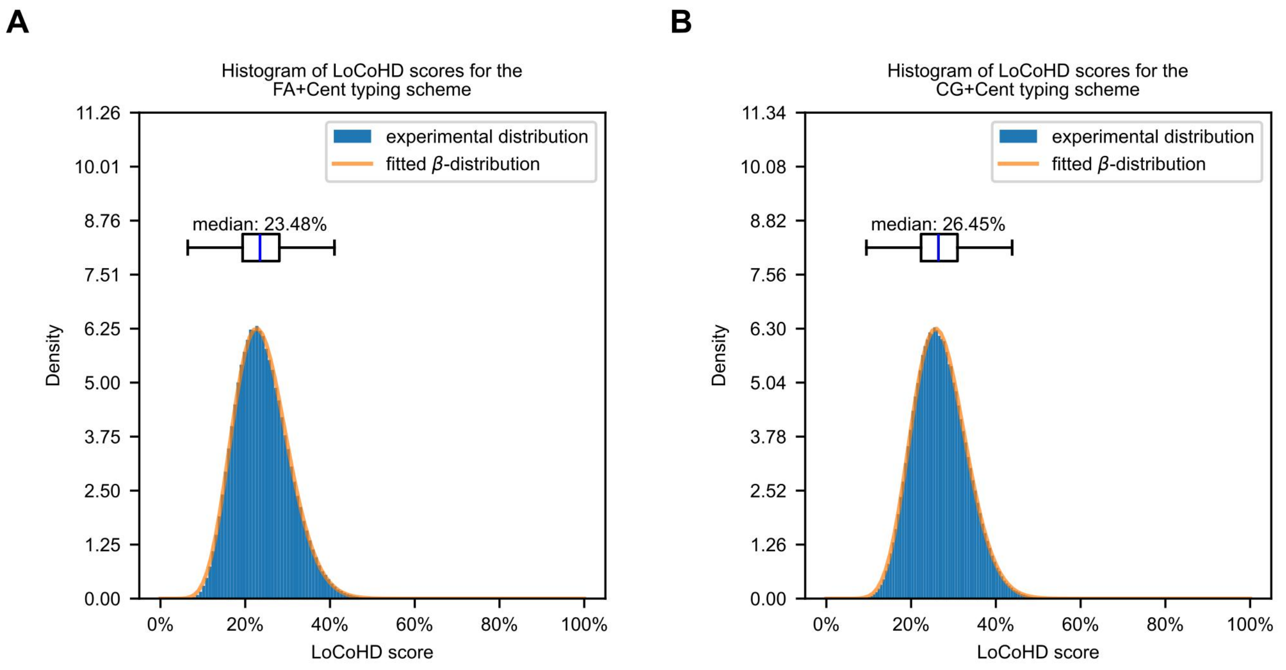

Supplementary Figure 1 Panel **A** corresponds to the histogram (“experimental distribution”, blue bars) of LoCoHD scores for the FA+Cent typing scheme, while Panel **B** corresponds to the histogram of LoCoHD scores for the CG+Cent typing scheme. Samples were generated by comparing the uncorrelated environments of random residue pairs. The uniform weight function between 3 Å and 10 Å was used. Both distributions can be modeled with high confidence using β-distributions (orange line plots). Box-and-whisker plots show the main statistical descriptors of the distributions: data ranges without outliers (whiskers), interquartile ranges (box edges) and the median (central blue line). Source data are provided as a Source Data file.

Supplementary Note 2: Average LoCoHD Score of Different Residue Category Pairs

To further demonstrate the discriminating ability of LoCoHD between environments of residues with different properties, we grouped residues based on their size, charge, hydrophobicity, aromaticity, and helix- and sheet-propensities. In each property category we separated residues into different sub-categories (e.g. in the category "size" we can differentiate the sub-categories "small", "medium-size", and "large"). On **Supplementary Figure 2** we plotted the average LoCoHD values for these sub-category pairs in case of primitive typing scheme FA+Cent, and on **Supplementary Figure 3** in the case of CG+Cent. It can be seen that in most of the cases the different sub-category pairs have significantly different average LoCoHD values.

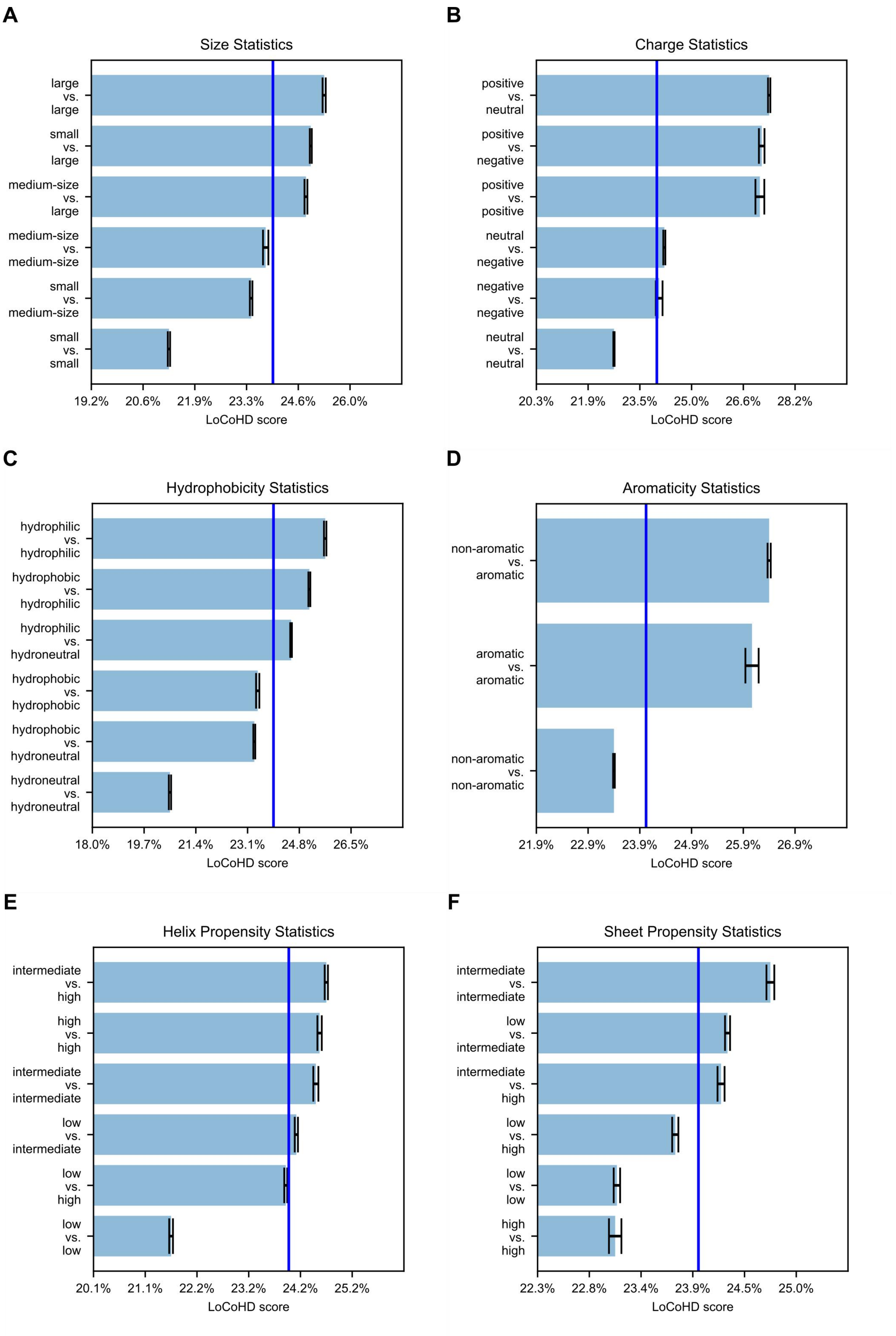

Supplementary Figure 2 showing the average LoCoHD scores of different residue category pairs (size of the blue bars) along with their two-sided, 95% confidence intervals (black error-bars). (**Continued on next page**)

Supplementary Figure 2 (Continuation) Data belongs to the primitive typing scheme FA+Cent and the uniform weight function between 3 Å and 10 Å. The vertical blue line indicates the overall average LoCoHD score. Panel **A** shows the residue size dependence of the average LoCoHD score. The one letter codes of the large residues are F, W, Y, I, L, M, K, R, medium-sized residues are V, H, E, Q, and small residues are C, P, T, D, N, A, G, S. Panel **B** shows the charge dependence of the average LoCoHD scores. Positive residues are R, K, negative residues are E, D, and neutral residues are the remaining ones. Panel **C** shows the hydrophobicity-dependence of the average LoCoHD scores. Here, E, D, K, R, Q, P, N are considered hydrophilic, H, A, T, S, V, G are considered hydroneutral, while M, C, I, L, Y, F, W are considered hydrophobic. Panel **D** shows how the average LoCoHD score depends on the residue pair's aromaticity. Aromatic residues are W, F, Y, H. Panel **E** shows a partitioning of residues based on their helix propensity. Residues with high helix propensity are A, L, E, Q, M, R, with intermediate propensity are V, I, F, Y, W, H, K, while with low propensity are G, S, T, D, N, C, P. The same partitioning can be seen on panel **F**, but now according to the beta sheet propensities. Residues with high beta sheet propensity are V, I, F, Y, C, W, with intermediate propensity are L, S, T, M, H, R, K, while with low propensity are G, A, D, E, N, Q, P. Source data are provided as a Source Data file.

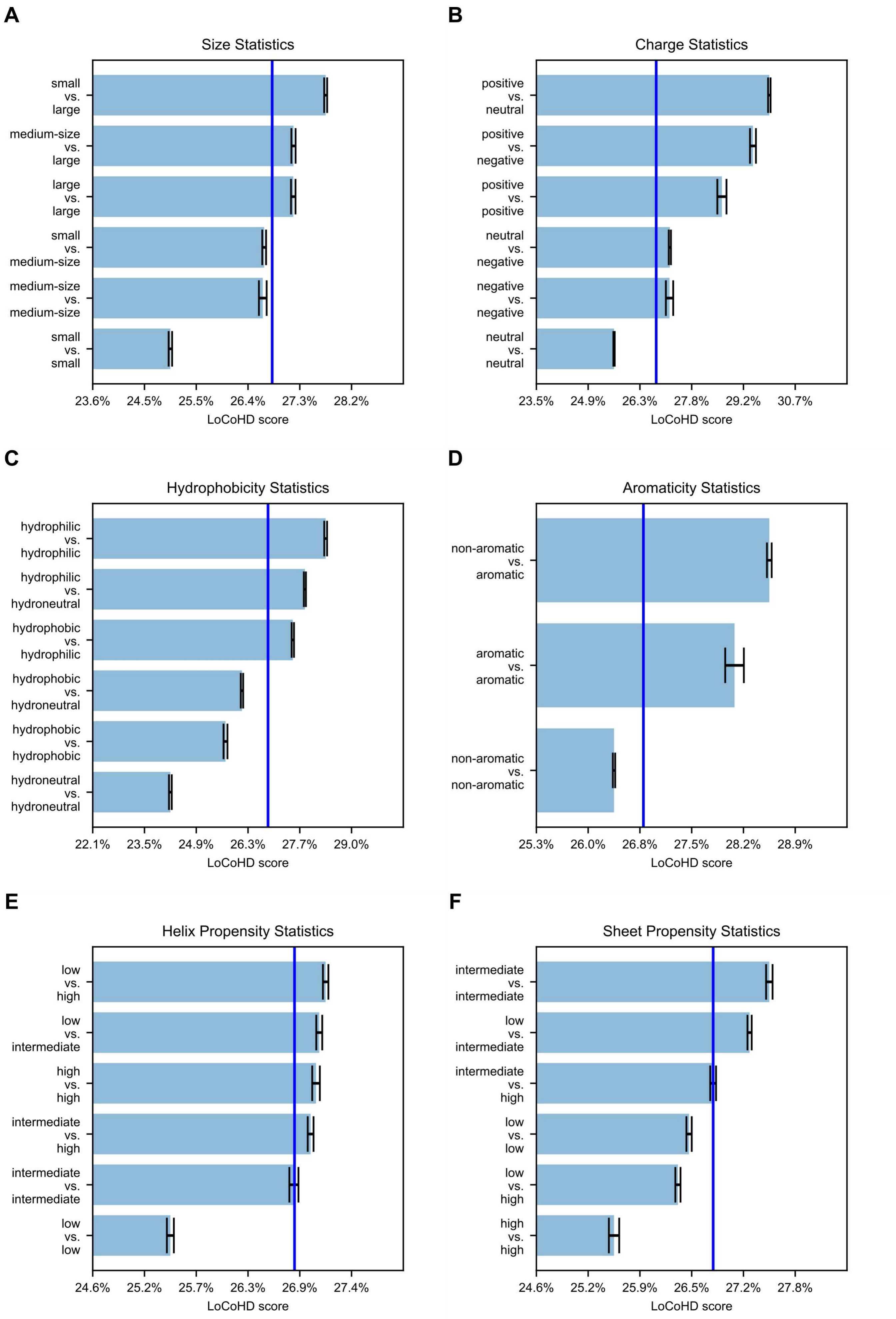

Supplementary Figure 3 showing the average LoCoHD scores of different residue category pairs (size of the blue bars) along with their two-sided, 95% confidence intervals (black error-bars). (**Continued on next page**)

Supplementary Figure 3 (Continuation) Data belongs to the primitive typing scheme CG+Cent and the uniform weight function between 3 Å and 10 Å. The vertical blue line indicates the overall average LoCoHD score. Panel **A** shows the residue size dependence of the average LoCoHD score. The one letter codes of the large residues are F, W, Y, I, L, M, K, R, medium-sized residues are V, H, E, Q, and small residues are C, P, T, D, N, A, G, S. Panel **B** shows the charge dependence of the average LoCoHD scores. Positive residues are R, K, negative residues are E, D, and neutral residues are the remaining ones. Panel **C** shows the hydrophobicity-dependence of the average LoCoHD scores. Here, E, D, K, R, Q, P, N are considered hydrophilic, H, A, T, S, V, G are considered hydroneutral, while M, C, I, L, Y, F, W are considered hydrophobic. Panel **D** shows how the average LoCoHD score depends on the residue pair's aromaticity. Aromatic residues are W, F, Y, H. Panel **E** shows a partitioning of residues based on their helix propensity. Residues with high helix propensity are A, L, E, Q, M, R, with intermediate propensity are V, I, F, Y, W, H, K, while with low propensity are G, S, T, D, N, C, P. The same partitioning can be seen on panel **F**, but now according to the beta sheet propensities. Residues with high beta sheet propensity are V, I, F, Y, C, W, with intermediate propensity are L, S, T, M, H, R, K, while with low propensity are G, A, D, E, N, Q, P. Source data are provided as a Source Data file.

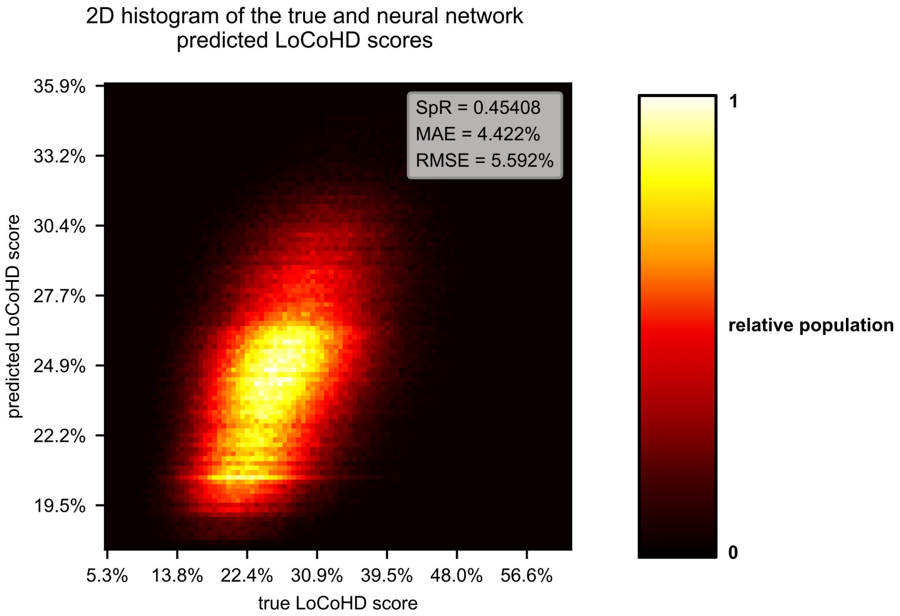

Supplementary Figure 4 Correlation-heatplot of the neural network predicted and true, target LoCoHD scores. Lighter colors mean more density in the region. The neural network was able to learn the connection between the secondary chemical interaction counts in the environment and the LoCoHD score of the environments with a mean average error (MAE) of 4.42%, a root mean squared error (RMSE) of 5.59% and a Spearman's correlation (SpR) of 0.454.

Supplementary Note 3 Comparison of Rat Trypsin Mutants with its Wild Type Structure

We compared the rat trypsin S195C mutant structure (PDB ID: 1DPO)[20], the rat trypsin D102N mutant structure (PDB ID: 2TRM)[21] and the wild type rat trypsin structure (PDB ID: 1ANE)[22]. The former mutation deactivates the catalytically active serine residue through a mutation to a cysteine, while the latter mutation is responsible for the functionality decrease of trypsin through the stabiliziation of His^{57} in an undesirable conformation. We used the FA+Cent typing scheme and the uniform weight function between 3 Å and 10 Å to compare all three proteins residue-by-residue, using the centroid primitive atoms as anchors.

Results can be seen in **Supplementary Figure 5** and **6**. Even though the three structures are almost identical (their backbone RMSD is below 0.3 Å), the structures shows high local chemical dissimilarity near the mutation-pair as identified by the LoCoHD metric. Both mutations cause large chemical alterations inside the environments: D102N removes two O_neg primitive types and adds an O_neu and an N_neu primitive atom, while S195C replaces an O_neu primitive type with an S primitive type. Besides the composition changes in the environment, some residues also undergo large conformational alterations. These effects - combined - cause some outlyingly high LoCoHD values. Examples are residues Arg^{96} and Gln^{192}, which have different conformations in different structures, as well as different environment primitive atom compositions.

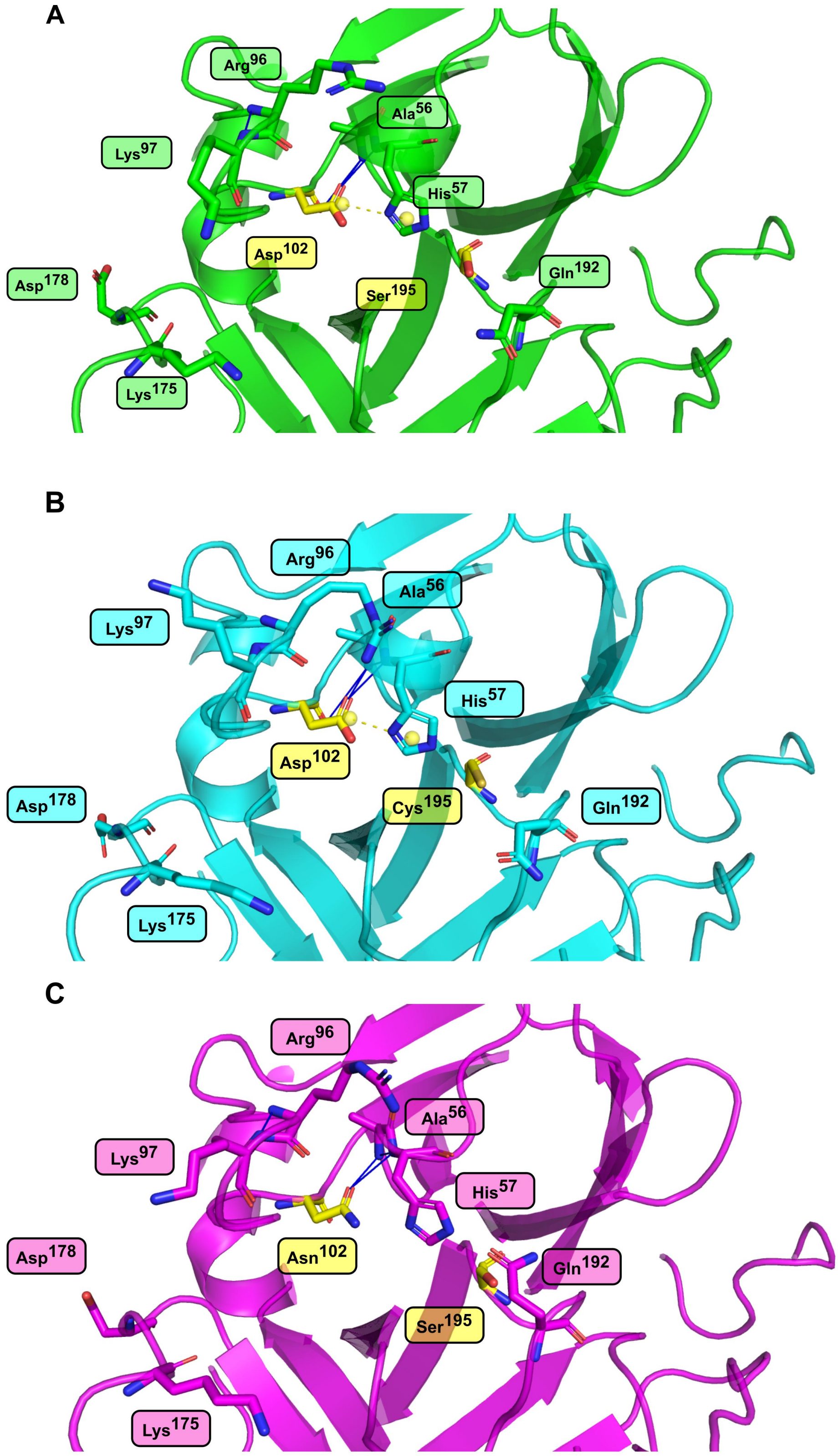

Supplementary Figure 5 Environments around sites 102 and 195 in the rat trypsin serine proteases. Panel **A** depicts the environment inside the wild type trypsin (green sticks and ribbons), panel **B** depicts the same for the S195C mutant (cyan sticks and ribbons), while panel **C** depicts the environment inside the D102N mutant (magenta sticks and ribbons). Residues with high LoCoHD scores are presented as stick models, namely Ala^{56}, His^{57}, Arg^{96}, Lys^{97}, Lys^{175}, Asp^{178} and Gln^{192}. Mutation sites are also highlighted as yellow stick models. Low opacity yellow spheres denote ionic interaction centers, yellow dashed lines denote ionic interactions and full blue lines denote H-bridges.

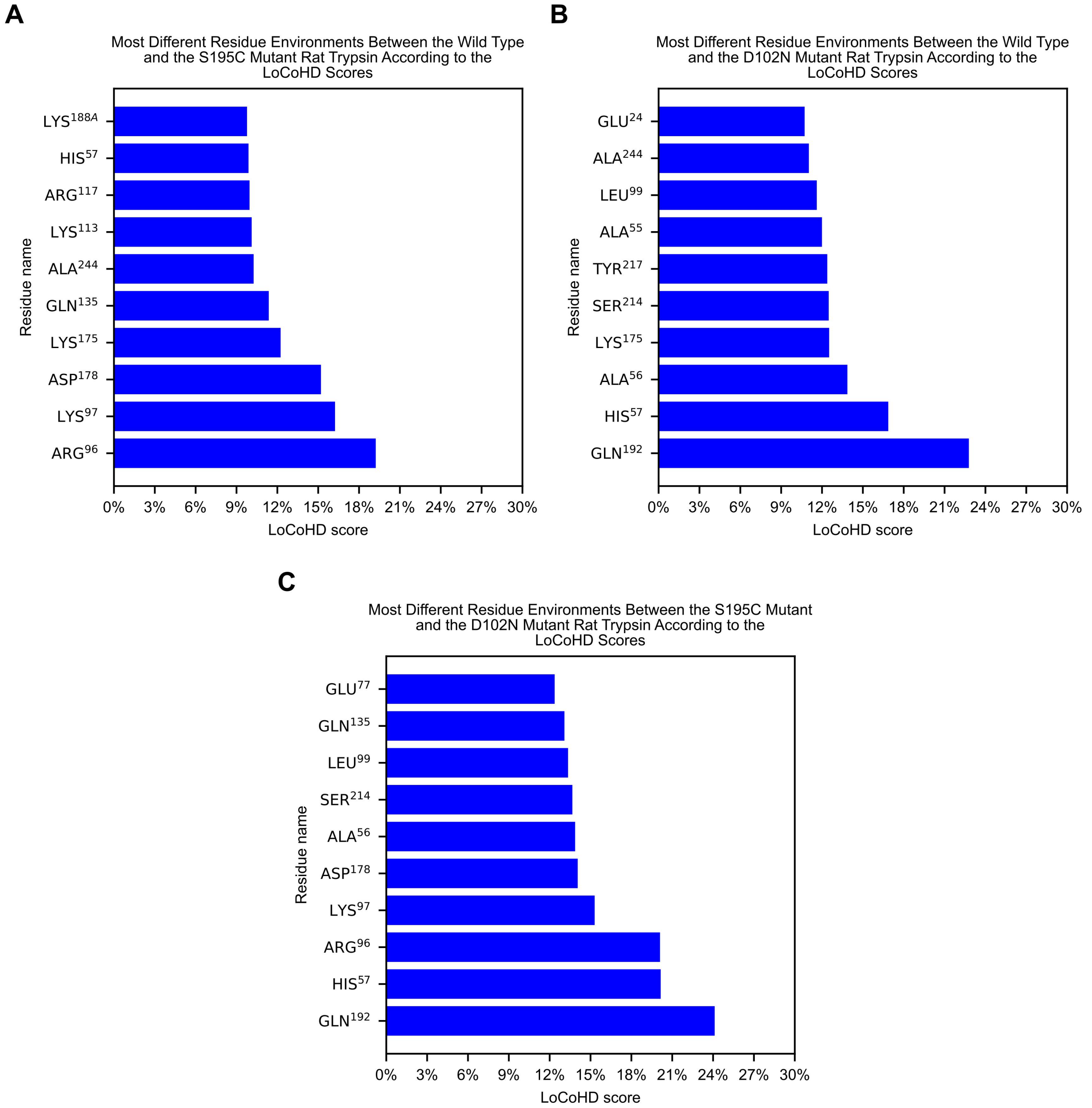

Supplementary Figure 6 Panels **A**, **B** and **C** collect LoCoHD comparisons of the different rat trypsin variants, namely the comparison of the wild type to the S195C mutant, the comparison of the wild type to the D102N mutant, and the comparison of the S195C mutant to the D102N mutant, respectively. Residues having the 10 largest LoCoHD scores are depicted on these diagrams along with their score values. Source data are provided as a Source Data file.

Supplementary Note 4 Comparison of three Aminoacyl Peptidase Variants

The structure of the wild type (PDB ID: 3O4G), D524N mutant (PDB ID: 3O4J) and D524A mutant (PDB ID: 3O4H) *Aeropyrum pernix* Aminoacyl Peptidase (ApAAP)[23] were investigated with LoCoHD. For this, the primitive typing scheme FA+Cent and the uniform weight function between 3 Å and 10 Å were used. This is a serine protease with the catalytic triad of Ser^{445}, Asp^{524} and His^{556}. The catalytic activity of the protein decreases significantly upon the introduction of either the mutation D524N (660-fold reduction) or the mutation D524A (2090-fold reduction). We found significant chemical changes on the surface of the enzyme and in its active site when the mutations were introduced. Results can be seen in **Supplementary Figure 7** and **8**.

Comparing the wild type with the D524N mutant, residues Arg^{345}, Glu^{498}, Arg^{501} and Gly^{33} get highlighted at the first four places. Arg^{345} participates in a salt-brigde with Glu^{339} in the wild type structure, while in the D524N mutant it forms a π-cation interaction with Trp^{337}. This change in the interaction partner is accompanied by a large conformational change of the arginine sidechain. Glu^{498} and Arg^{501} are both in the same environment, for which the large LoCoHD can be attributed to the conformational change of Arg^{501}, shifting its guadinio group away from Glu^{475} and towards Glu^{498}. Gly^{33} is inside a loop element in both structures. The two glycines appear in two different conformational states: in 3O4G the (φ, ψ) angle pair for Gly^{33} is (-175°, -80°), while in 3O4J it is (174°, 70°). The effect of the latter conformation is the closeness of the Asp^{32} carboxylate to the glycine backbone NH atom, i.e. the formation of a sidechain-backbone H-bond, an interaction which is not present in 3O4G. Interestingly, locations close to the active site only appear on the 6th (His^{556}) and 7th (Thr^{527}) places, where the direct effect of the mutation causes the detected environmental changes.

The introduction of the D524A is more dramatic when the LoCoHD scores are considered. Residues His^{556} and Tyr^{444} both overtake Arg^{345}, which are two residues close to the active site. Tyr^{444} undergoes a dramatic conformational change, shifting its aromatic sidechain away from the catalytic histidine. This lowers the aromatic carbon (C_aro primitive type) content directly around the centroid of His^{556}, an effect which is detected through the LoCoHD score. Thr^{527} and Phe^{381} also appear on the top 10 LoCoHD score list and are close to the active site. The sidechain rotation of Tyr^{444} introduces an edge-to-face π-π interaction with Phe^{381}.

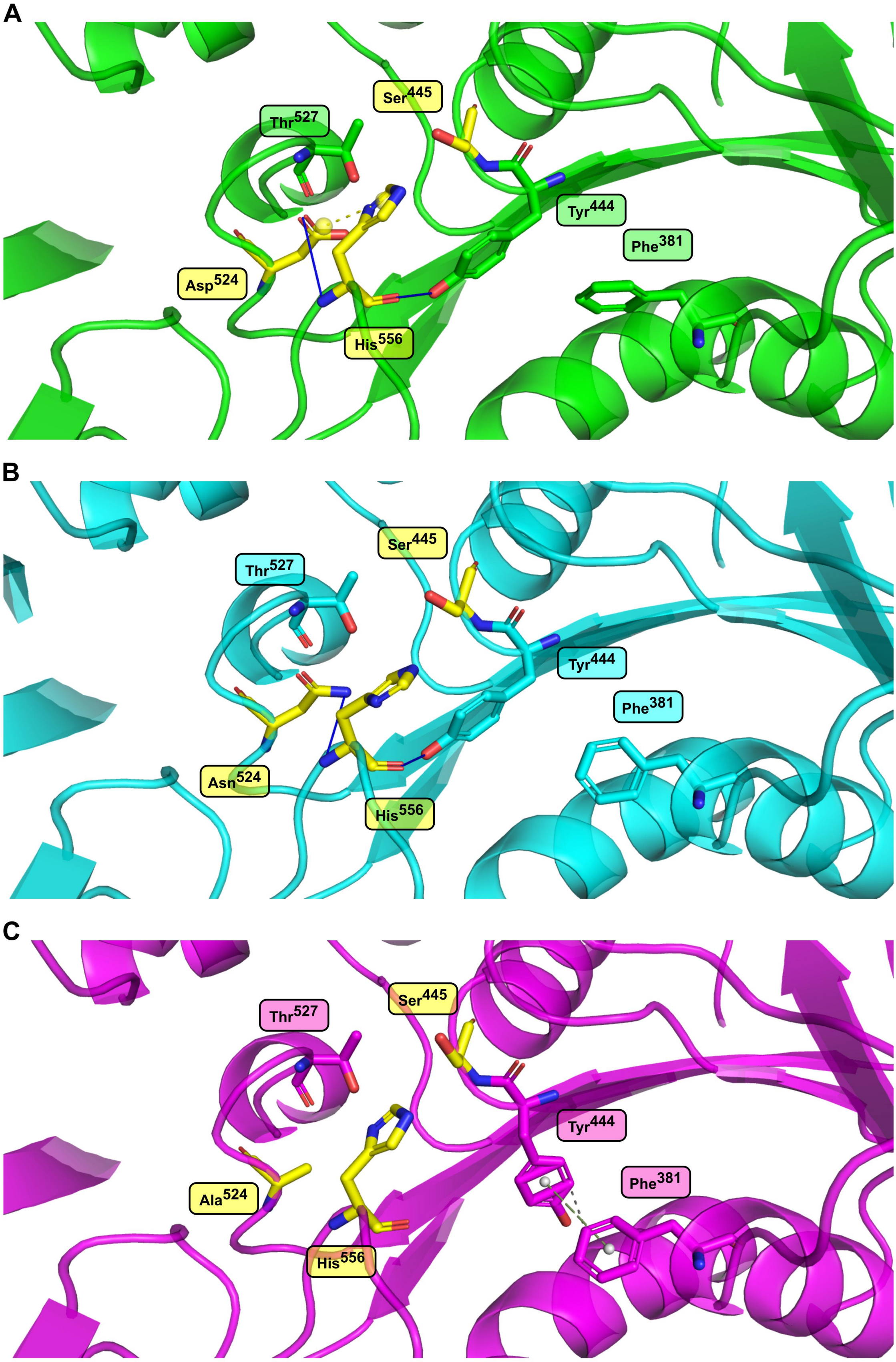

Supplementary Figure 7 Panels **A**, **B** and **C** show environments around the ApAAP active site in the wild type protein (green sticks and ribbons), in the D524N (cyan sticks and ribbons) and in the D524A (magenta sticks and ribbons) mutants, respectively. The catalytic residues in the active site are Ser^{445}, Asp^{524} and His^{556}. Active site residues which undergo large chemical environmental changes indicated by LoCoHD (Phe^{381}, Tyr^{444}, Thr^{527}) are highlighted with stick representations. Low opacity yellow spheres denote ionic interaction centers, yellow dashed lines denote ionic interactions, full blue lines denote H-bridges, gray spheres denote pi interaction centers, long-dashed gray lines denote pi-pi interactions and short-dashed gray lines denote Van der Waals contacts.

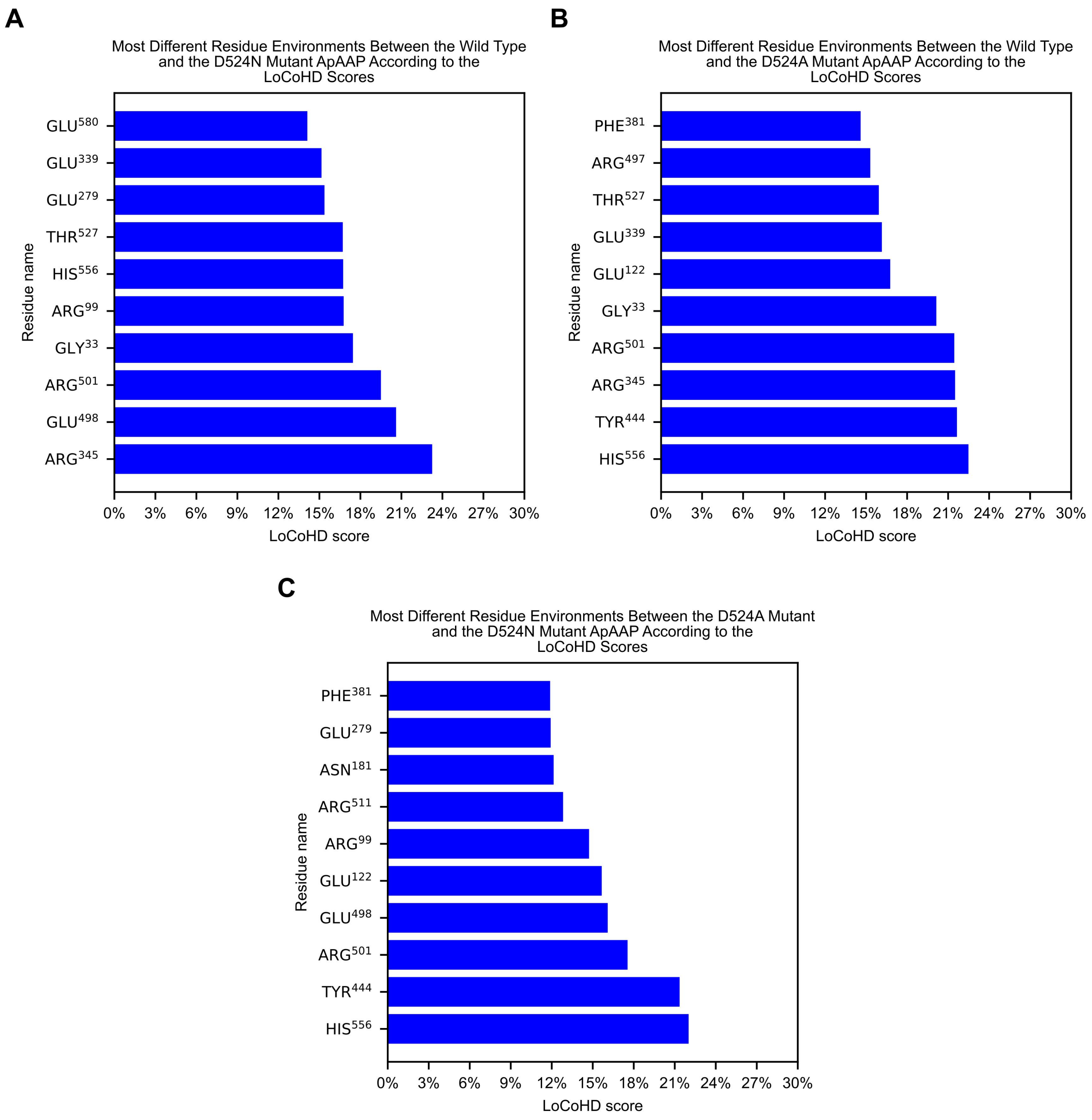

Supplementary Figure 8 Panels **A**, **B** and **C** collect LoCoHD comparisons of the different ApAAP variants, namely the comparison of the wild type to the D524N mutant, the comparison of the wild type to the D524A mutant, and the comparison of the D524N mutant to the D524A mutant, respectively. Residues having the 10 largest LoCoHD scores are depicted on these diagrams along with their score values. Source data are provided as a Source Data file.

	AlphaFold2 (TS427)	BAKER (TS473)	BAKER-experimental (TS403)	FEIG-R2 (TS480)	Zhang (TS129)
mean SpR(lDDT, LoCoHD)	-0.6534	-0.5288	-0.5162	-0.4773	-0.4581
median SpR(lDDT, LoCoHD)	-0.6788	-0.5550	-0.5257	-0.5071	-0.4847
StDev SpR(lDDT, LoCoHD)	0.1106	0.1239	0.1279	0.1632	0.1583
min SpR(lDDT, LoCoHD)	-0.8468	-0.7505	-0.7505	-0.7851	-0.7485
max SpR(lDDT, LoCoHD)	-0.2961	-0.1476	-0.0602	0.0146	-0.0690
mean SpR(CAD, LoCoHD)	-0.6259	-0.5283	-0.5087	-0.4788	-0.4703
median SpR(CAD, LoCoHD)	-0.6297	-0.5540	-0.5180	-0.4843	-0.4786
StDev SpR(CAD, LoCoHD)	0.1177	0.1136	0.1183	0.1373	0.1309
min SpR(CAD, LoCoHD)	-0.8089	-0.7396	-0.7185	-0.7776	-0.7772
max SpR(CAD, LoCoHD)	-0.1343	-0.1813	-0.0854	-0.1261	-0.1353
mean prm-lDDT	0.8210	0.5744	0.5688	0.5156	0.5084
median prm-lDDT	0.8410	0.6000	0.5860	0.5350	0.5210
StDev prm-lDDT	0.0884	0.1344	0.1304	0.1471	0.1347
min prm-lDDT	0.4590	0.2400	0.2420	0.2020	0.1985
max prm-lDDT	0.9320	0.7845	0.7860	0.7505	0.7510
mean prm-CAD	0.7756	0.5698	0.5675	0.5269	0.5140
median prm-CAD	0.7920	0.5910	0.5850	0.5340	0.5280
StDev prm-CAD	0.0731	0.1159	0.1064	0.1157	0.1094
min prm-CAD	0.4495	0.2775	0.3080	0.2735	0.2630
max prm-CAD	0.8710	0.7510	0.7760	0.7130	0.7330
mean prm-LoCoHD	0.0831	0.1316	0.1338	0.1425	0.1472
median prm-LoCoHD	0.0814	0.1311	0.1340	0.1425	0.1482
StDev prm-LoCoHD	0.0177	0.0216	0.0211	0.0215	0.0213
min prm-LoCoHD	0.0529	0.0829	0.0811	0.0984	0.0951
max prm-LoCoHD	0.1457	0.1904	0.1957	0.1860	0.2028

Supplementary Table 2 lDDT, CAD and LoCoHD scoring statistics for the first five CASP14 contestants. For each descriptor (SpR, per-residue median (prm-) lDDT, prm-CAD, prm-LoCoHD) the mean, median, standard deviation (StDev), minimum, and maximum values are reported over all datasets, i.e. protein structures. It can be observed, that the lDDT and LoCoHD scoring systems agree on the order of the five contestants (rows median prm-lDDT and median prm-LoCoHD). Also, it can be seen that as the quality of the prediction decreases (lDDT decreases, LoCoHD increases) the magnitude of the median SpR value decreases (row median SpR(lDDT, LoCoHD)). The same behavior can be observed for the relationship between the CAD and LoCoHD scores. Source data are provided as a Source Data file.

largest gap of

	SpR (lDDT, LoCoHD)	SpR (CAD, LoCoHD)	prm-LoCoHD	prm-lDDT	prm-CAD
**AlphaFold2 (TS427)**	0.3512 (T1064_1 vs. 5)	0.2805 (T1064_1 vs. 5)	0.0392 (T1064_1 vs. 5)	0.1945 (T1064_1 vs. 5)	0.1390 (T1064_1 vs. 5)
**BAKER (TS473)**	0.3451 (T1080_1 vs. 4)	0.5051 (T1043_2 vs. 5)	0.0482 (T1042_1 vs. 5)	0.3265 (T1042_1 vs. 5)	0.2880 (T1054_1 vs. 5)
**BAKER-experimental (TS403)**	0.2673 (T1033_1 vs. 4)	0.2120 (T1080_2 vs. 4)	0.0457 (T1037_1 vs. 5)	0.3500 (T1037_1 vs. 5)	0.2250 (T1037_1 vs. 5)
**FEIG-R2 (TS480)**	0.2550 (T1080_1 vs. 4)	0.1996 (T1046s_1 vs. 3)	0.0311 (T1046s_1 vs. 5)	0.1020 (T1082_1 vs. 2)	0.1190 (T1082_1 vs. 2)
**Zhang (TS129)**	0.3671 (T1031_2 vs. 5)	0.2931 (T1082_4 vs. 5)	0.0425 (T1026_1 vs. 3)	0.2450 (T1026_1 vs. 4)	0.2260 (T1026_1 vs. 4)

Supplementary Table 3 Showing the largest gap sizes for the different measures (Spearman's correlation coefficient (SpR) and per-residue median (prm) scores). These gap sizes belong to one single target structure's predictions (the target structure's code is indicated between the brackets). Gap sizes are calculated by subtracting the minimum value from the maximum value belonging to the target structure in question. The prediction numbers (after the target structure IDs and the underscore) creating the largest gaps are also indicated within the brackets. Source data are provided as a Source Data file.

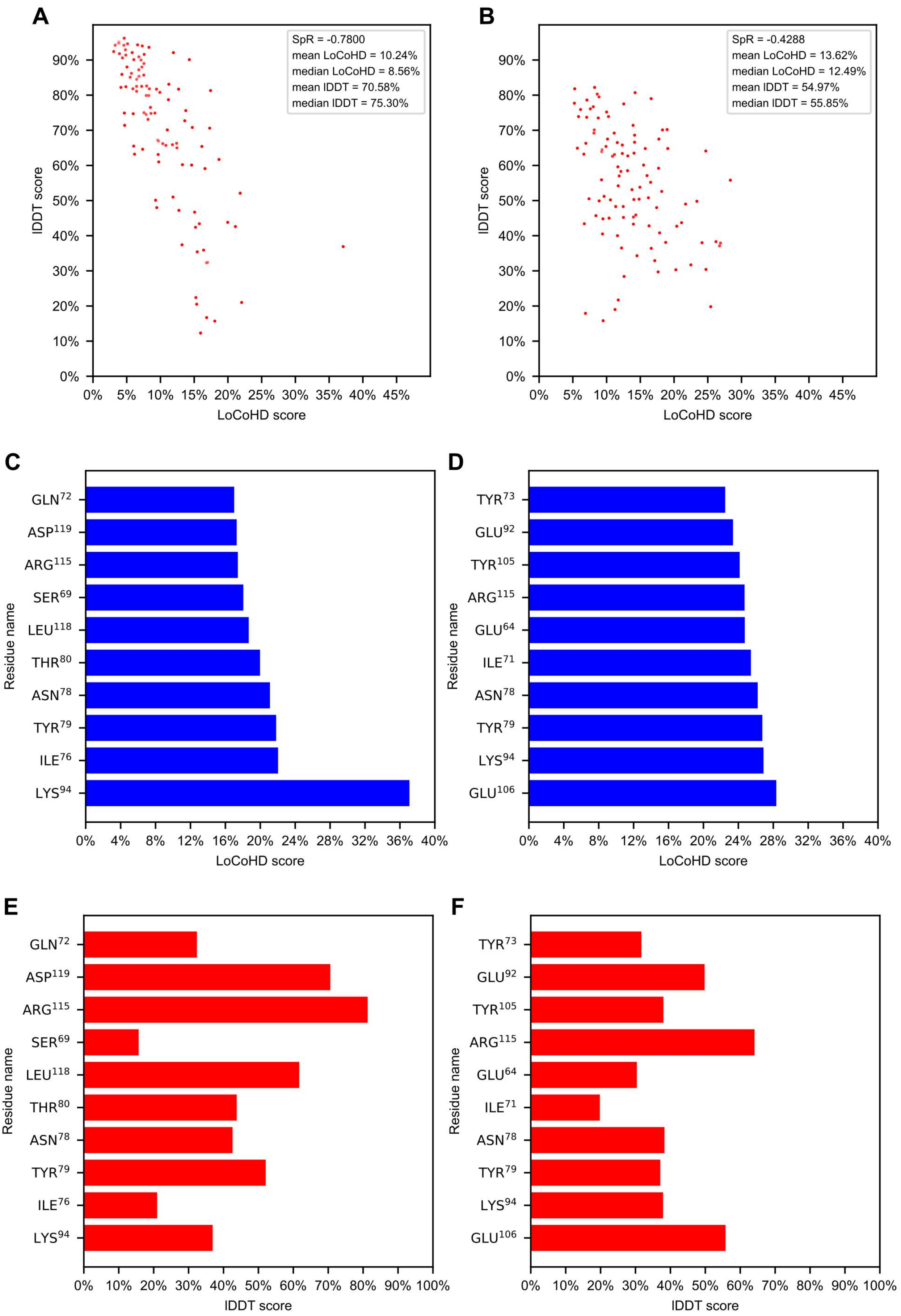

Supplementary Figure 9 lDDT-LoCoHD correlation scatter plots (panels **A** and **B**) and the LoCoHD (panels **C** and **D**) and lDDT (panels **E** and **F**) values of the residues with the ten highest LoCoHD scores. The plots correspond to the AlphaFold2 predicted structures T1064TS427_1 (panels **A**, **C** and **E**) and T1064TS427_5 (panels **B**, **D** and **F**) compared to the experimental structure T1064 (SARS-CoV-2 ORF8 accessory protein). Source data are provided as a Source Data file.

	Yang-Server (TS229)	PEZY Foldings (TS278)	Yang (TS439)	DFolding (TS074)
number of analyzable reference structures	17	41	38	12
mean SpR(lDDT, LoCoHD)	-0.6675	-0.6588	-0.6595	-0.6590
median SpR(lDDT, LoCoHD)	-0.7126	-0.6842	-0.6838	-0.7187
StDev SpR(lDDT, LoCoHD)	0.1253	0.1282	0.1084	0.1377
min SpR(lDDT, LoCoHD)	-0.8365	-0.8451	-0.8511	-0.7917
max SpR(lDDT, LoCoHD)	-0.2808	-0.0785	-0.2720	-0.2496
mean SpR(CAD, LoCoHD)	-0.6440	-0.6478	-0.6347	-0.6049
median SpR(CAD, LoCoHD)	-0.7020	-0.6778	-0.6467	-0.6879
StDev SpR(CAD, LoCoHD)	0.1441	0.1363	0.1201	0.1752
min SpR(CAD, LoCoHD)	-0.8077	-0.8220	-0.8324	-0.7834
max SpR(CAD, LoCoHD)	-0.2227	-0.0342	-0.1798	-0.1343
mean prm-lDDT	0.7978	0.8007	0.8355	0.6783
median prm-lDDT	0.8605	0.8380	0.8755	0.7615
StDev prm-lDDT	0.1544	0.1185	0.1274	0.1898
min prm-lDDT	0.4260	0.4210	0.4295	0.3210
max prm-lDDT	0.9490	0.9335	0.9530	0.9070
mean prm-CAD	0.7640	0.7600	0.7858	0.6685
median prm-CAD	0.8080	0.7870	0.8150	0.7170
StDev prm-CAD	0.1140	0.1002	0.1051	0.1364
min prm-CAD	0.4775	0.4120	0.4395	0.4480
max prm-CAD	0.8910	0.8750	0.8960	0.8390
mean prm-LoCoHD	0.0738	0.0821	0.0725	0.0931
median prm-LoCoHD	0.0733	0.0794	0.0686	0.0892
StDev prm-LoCoHD	0.0147	0.0175	0.0179	0.0216
min prm-LoCoHD	0.0535	0.0485	0.0463	0.0601
max prm-LoCoHD	0.1161	0.1359	0.1330	0.1417

Supplementary Table 4 lDDT, CAD and LoCoHD scoring statistics for some of the best performing CASP15 contestants. For each descriptor (SpR, per-residue median (prm-) lDDT, prm-CAD, prm-LoCoHD) the mean, median, standard deviation (StDev), minimum, and maximum values are reported over all datasets, i.e. protein structures.

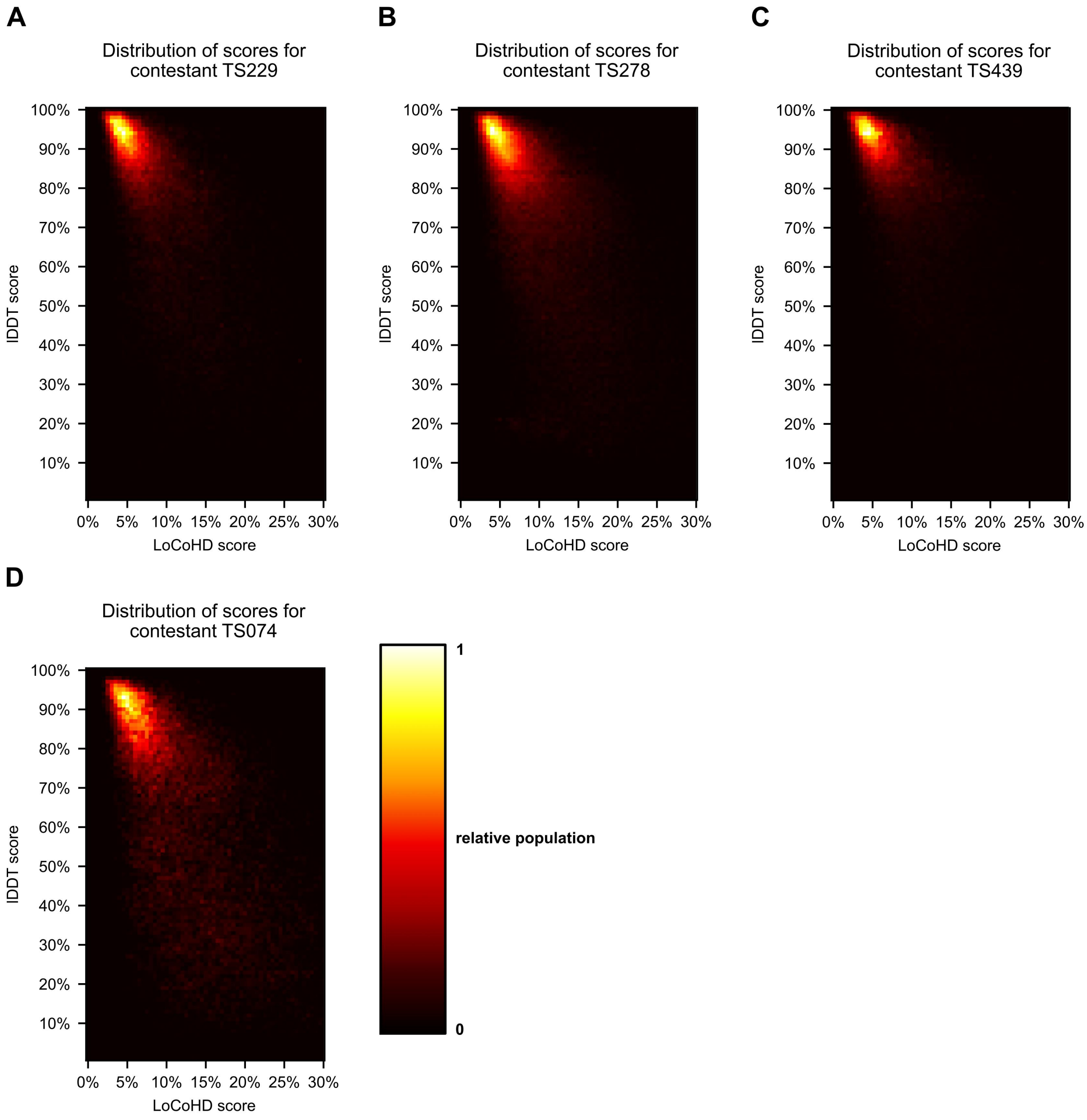

Supplementary Figure 10 Visualizing the two-dimensional distributions of the per-residue LoCoHD-lDDT pairs for each CASP15 contestant examined. On panel A, B, C, and D the histograms for the structure predictors Yang-Server, PEZYFoldings, Yang and DFolding can be seen, respectively. Histograms are depicted as heat maps, with warmer colors indicating higher populations in the corresponding area.

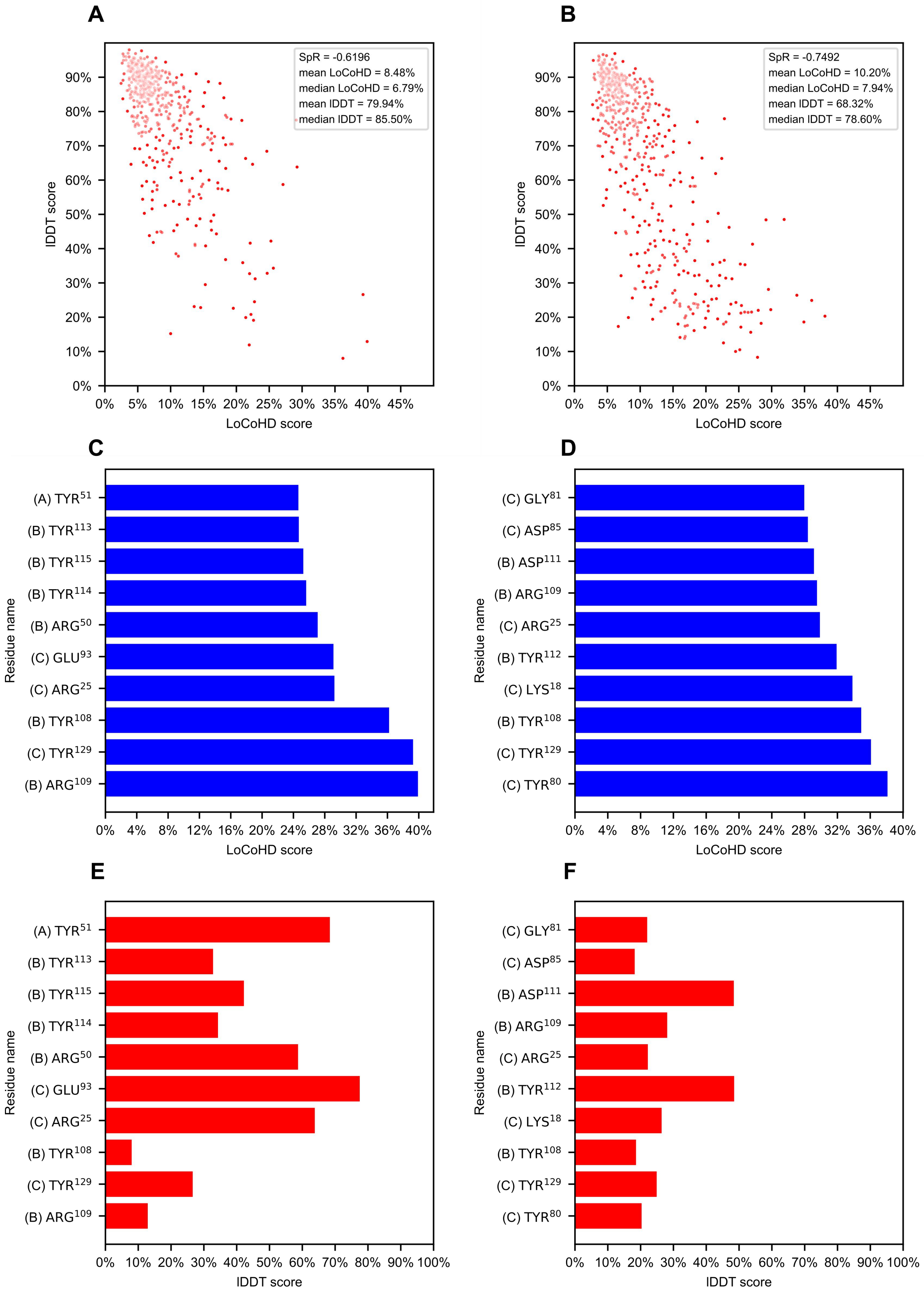

Supplementary Figure 11 lDDT-LoCoHD correlation scatter plots (panels **A** and **B**) and the LoCoHD (panels **C** and **D**) and lDDT (panels **E** and **F**) values of the residues with the ten highest LoCoHD scores (characters in parentheses denote chain names according to the target structure's nomenclature). The plots correspond to the (CASP15 contestant) PEZYFoldings predicted structures H1166TS278_1 (panels **A**, **C** and **E**) and H1166TS278_5 (panels **B**, **D** and **F**) compared to the experimental structure H1166 (human Fab S24-188 in complex with the N-terminal domain of the SARS-CoV-2 Nucleocapsid protein).

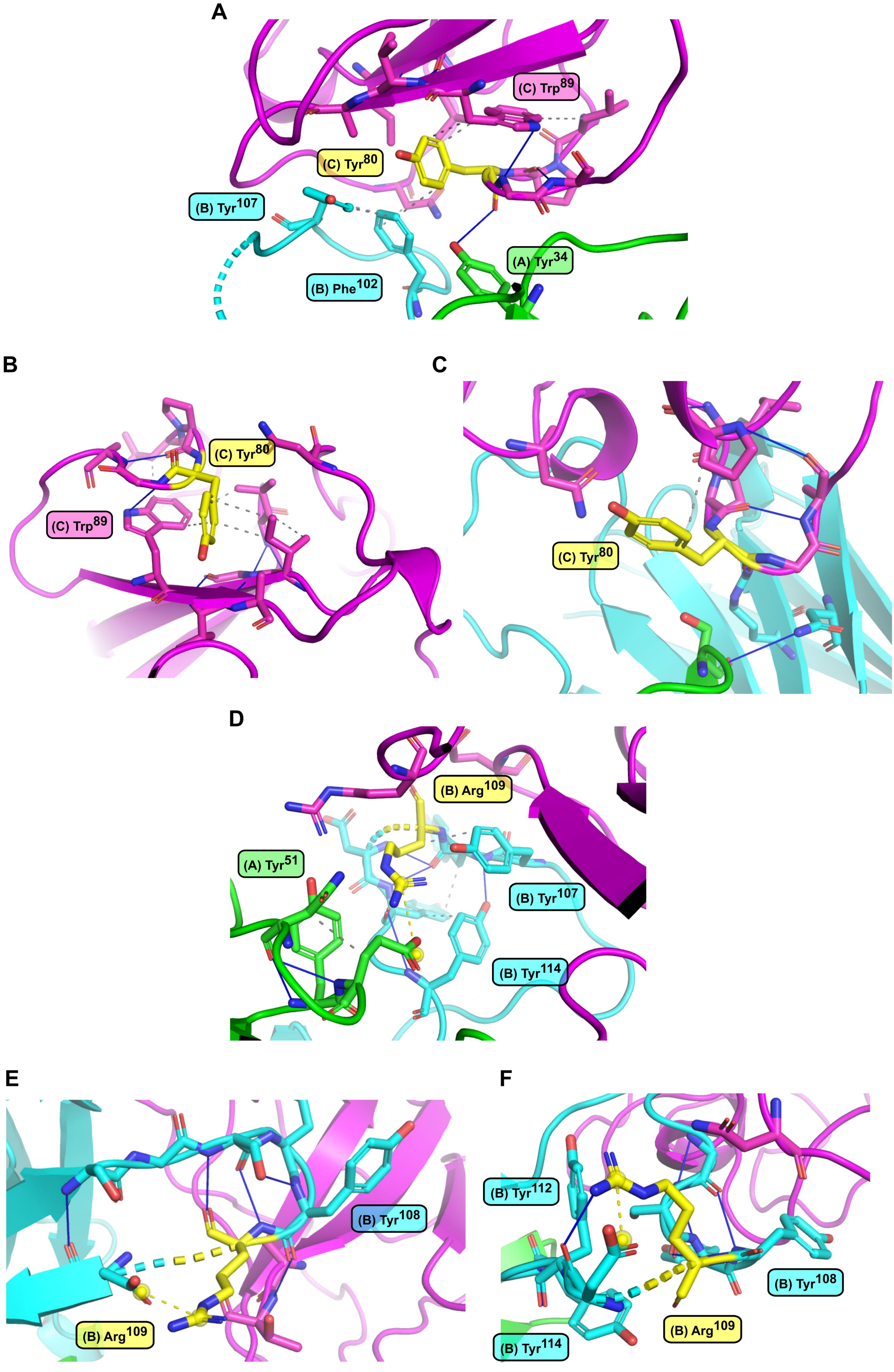

Supplementary Figure 12 High LoCoHD residue environments in the CASP15 PEZYFoldings predicted structures H1166TS278_1 and H1166TS278_5. On panels **A** and **D** the experimental structure can be seen, on panels **B** and **E** environments from H1166TS278_1 are visible, while on panels **C** and **F** structural snippets from H1166TS278_5 can be observed. (**Continued on next page**)

Supplementary Figure 12 (Continuation) Panels **A**, **B** and **C** show the environment of the residue Tyr^{80} from chain C (the antigen). Panels **D**, **E** and **F** show the environment of the residue Arg^{109} from chain B (the antibody). In both cases, the large LoCoHD relative to the experimental structure can be explained due to the aromatic primitive type content changes in the environments. In the true structure, Tyr^{80} is surrounded by several other aromatic partners, both from the antibody side and the antigenic side. In the predicted structure H1166TS278_1 only Trp^{89} from chain C remains as an interacting partner, while in H1166TS278_5 the difference is more dramatic, since no surrounding aromatic residues can be observed. For Arg^{109} from chain B the case is similar: in the experimental structure three aromatic residues constitute it's environment. This is not reproduced in H1166TS278_1, but is somewhat similar in H1166TS278_5. Low opacity yellow spheres denote ionic interaction centers, yellow dashed lines denote ionic interactions, full blue lines denote H-bridges, and short-dashed gray lines denote Van der Waals contacts.

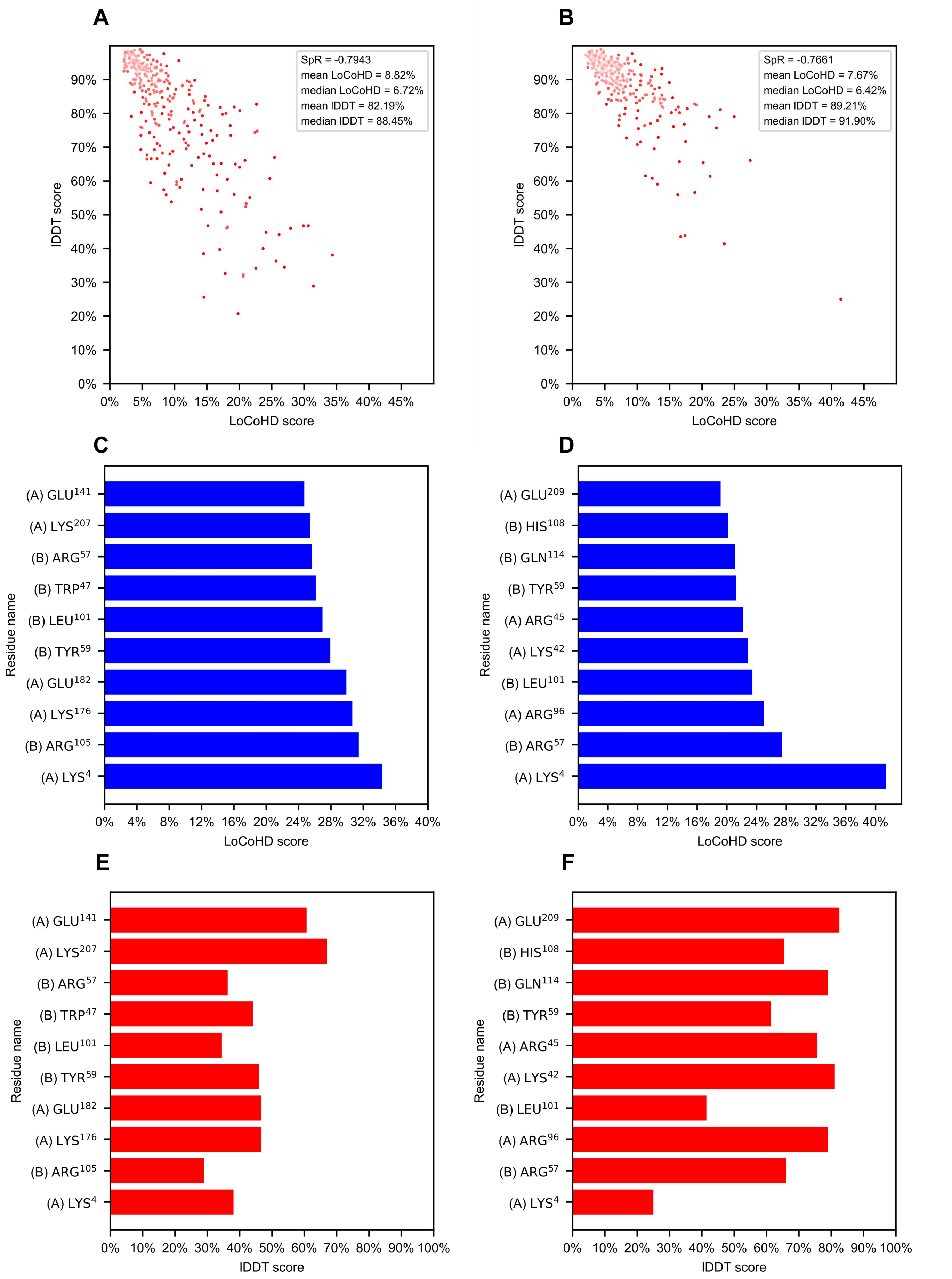

Supplementary Figure 13 lDDT-LoCoHD correlation scatter plots (panels **A** and **B**) and the LoCoHD (panels **C** and **D**) and lDDT (panels **E** and **F**) values of the residues with the ten highest LoCoHD scores (characters in parentheses denote chain names according to the target structure's nomenclature). The plots correspond to the (CASP15 contestant) PEZYFoldings predicted structures H1144TS278_1 (panels **A**, **C** and **E**) and H1144TS278_5 (panels **B**, **D** and **F**) compared to the experimental structure H1144 (mouse/alpaca CNPase-Nb8d nanobody-antigen complex).

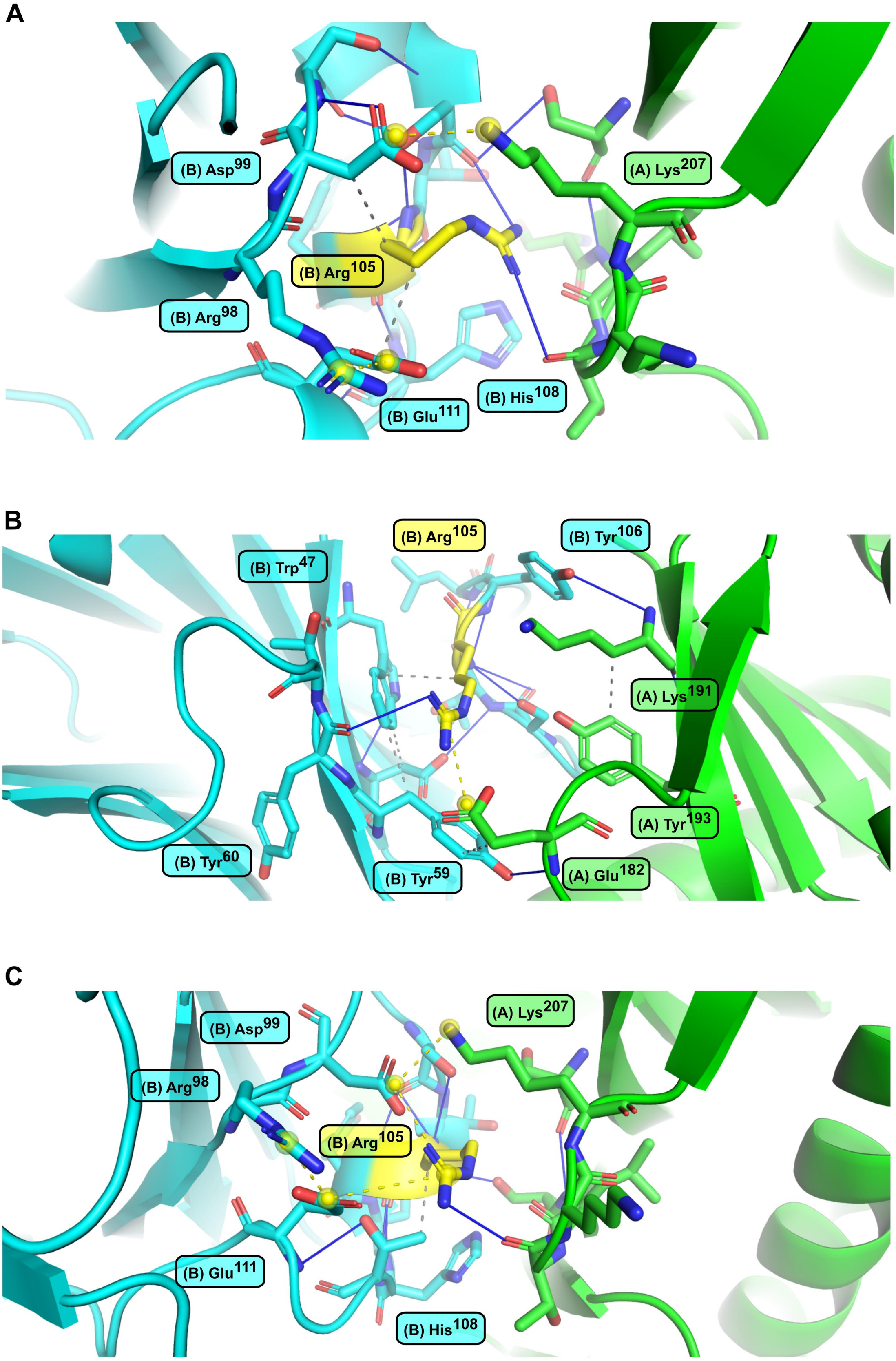

Supplementary Figure 14 High LoCoHD residue environments in the CASP15 PEZYFoldings predicted structures H1144TS278_1 and H1144TS278_5 (panels **B** and **C**), and in the experimental structure (panel **A**) around residue Arg^{105} chain B. Chain A (depicted with green) is the antigen (mouse CNPase), while chain B is the nanobody (alpacca Nb8d). Arg^{105} from chain B has the second largest LoCoHD after the N-terminal Lys^{4} (chain A) in H1144TS278_1, but it does not appear in the list of the top10 largest LoCoHD residues of H1144TS278_5. Looking at the structures, it is evindent that the chemical composition of the environment of Arg^{105} in H1144TS278_5 mostly resembles the composition of the same environment in the experimental structure, but this environment is very different in H1144TS278_1. This contrast in the two predicted structures arises due to the incorrectly docked nanobody position in H1144TS278_1.

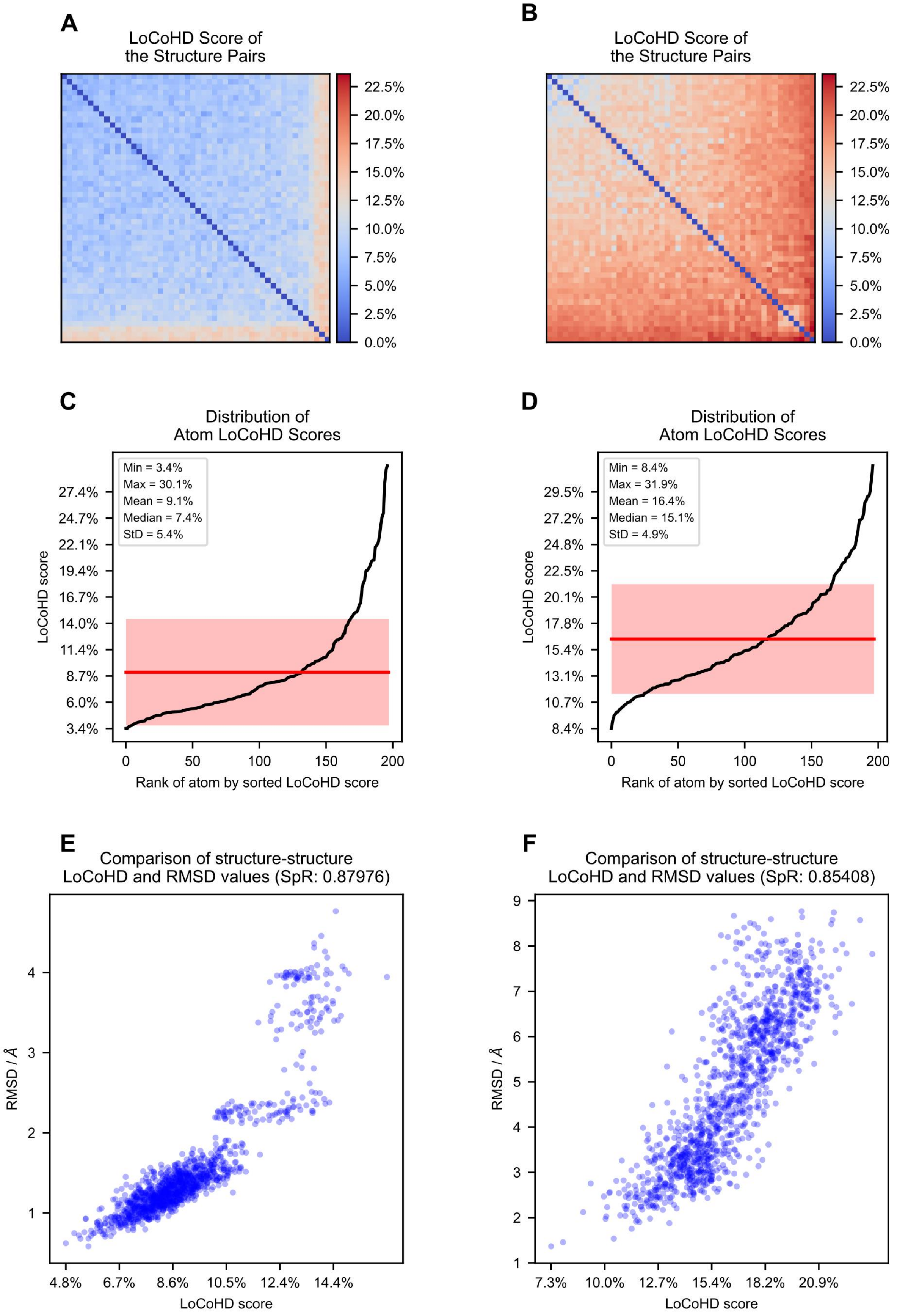

Supplementary Figure 15 LoCoHD and RMSD measurements for the E5 NMR ensemble. Panels **A**, **C** and **E** correspond to the ensemble at a temperature of 277K, while panels **B**, **D** and **F** correspond to the ensemble at 321K. Panels **A** and **B** show the 50 by 50 structure-structure LoCoHD distance matrix of the ensembles. Panels **C** and **D** show the average LoCoHD of each primitive atom arranged in an increasing order, along with some statistical descriptors. Panels **E** and **F** show the LoCoHD score, as well as the RMSD of every possible structure-structure comparison within the 50 member ensemble. The Spearman's correlation coefficients are also presented. Source data are provided as a Source Data file.

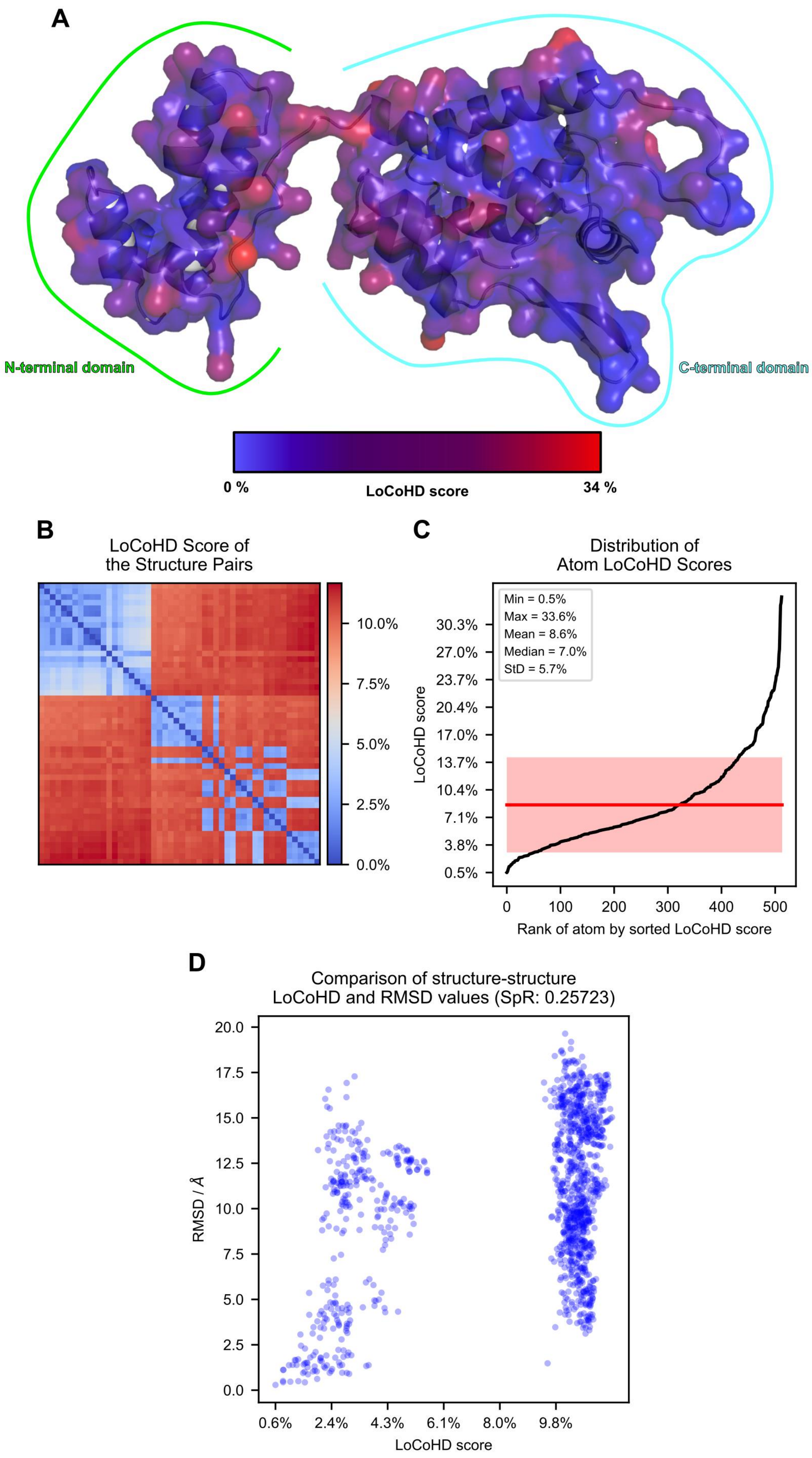

Supplementary Figure 16 LoCoHD and RMSD measurements for the HIV-1 Gag-Pol ensemble. Panel **A** shows a structure exemplar from the ensemble, with a surface colored according to the average LoCoHD score of the corresponding surface primitive atoms. Panel **B** shows the structure-structure primitive-atom-average LoCoHD comparison matrix of the ensemble. Panel **C** shows the structure-structure average LoCoHD scores of each primitive atom in an increasing order, along with some statistical descriptors. Panel **D** shows the LoCoHD score, as well as the RMSD of every possible structure-structure comparison within the 50 member ensemble. The Spearman's correlation coefficients are also presented. Source data are provided as a Source Data file.

residue	primitive atom name	LoCoHD score
Arg^{150}	Pos	33%
Arg^{214}	Pos	30%
Arg^{229}	Pos	24%
Met^{276}	Sulf	24%
Tyr^{277}	Aro	27%
Tyr^{277}	OH	34%
Ser^{278}	OH	27%
Thr^{280}	OH	25%
Ser^{281}	OH	24%
Arg^{299}	Pos	33%
Lys^{302}	Pos	25%
Arg^{305}	Pos	25%

Supplementary Table 5 Residues having the highest average LoCoHD score bearing primitive atoms in the HIV-1 Gag-Pol ensemble. Source data are provided as a Source Data file.

Supplementary Note 5: LoCoHD Scores for the Gamma Subunit of cGMP Phosphodiesterase

We also investigated the NMR-ensemble of the gamma subunit of cGMP phosphodiesterase (PED ID: PED00075, reference UniProt ID: P04972, PDB ID: 2JU4)[24], the result of which can be seen on **Supplementary Figure 17**. Again, we used the same CG primitive typing scheme as in the case of the HIV-1 Gag-Pol protein. This ensemble is of an 87 residue long intrinsically disordered protein (IDP), which contrasts well with the previously presented examples. The SpR between the LoCoHD and RMSD scores is -0.08, indicating no correlation between these metrics. The LoCoHD scores of the structure-structure comparisons range almost uniformly between approximately 19% and 25%, while the RMSD values range between 6 Å and 19 Å. Primitive atoms with the largest average LoCoHD values (>32%) include Arg^{11} Pos, Lys^{39} Pos, Lys^{41} Pos, and Glu^{77} Neg.

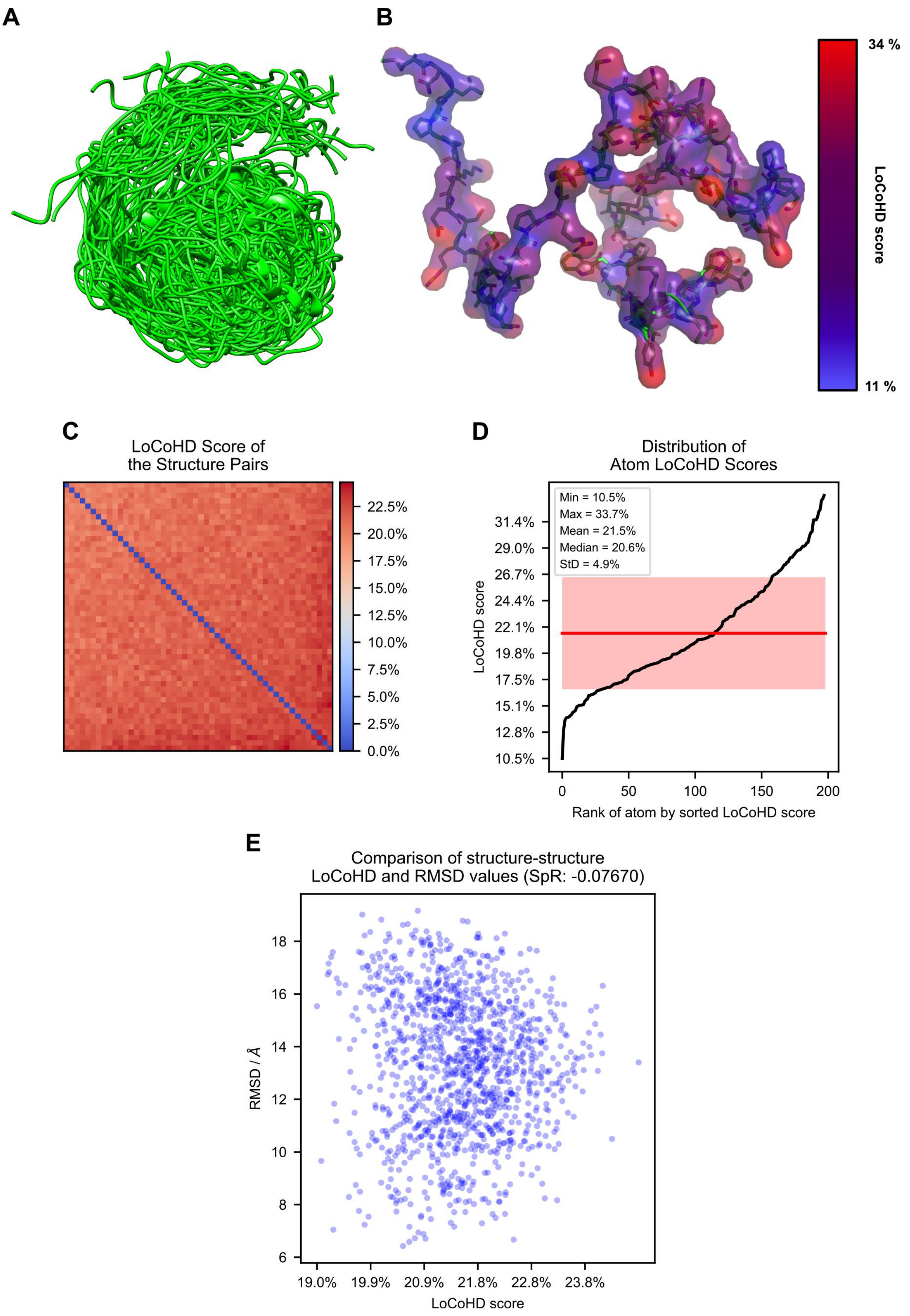

Supplementary Figure 17 Panels **A** and **B** are different depictions of the gamma subunit of cGMP phosphodiesterase, which is an intrinsically disordered protein. On panel **A**, the ensemble of structures are shown aligned together. It can be seen, that the alignment is of low quality, due to the high structural dissimilarity of the ensemble elements. On panel **B**, one element of the ensemble is shown, colored according to the ensemble average of the primitive atomic LoCoHD scores. Panels **C** and **D** depict analysis similar to that of the E5 and the HIV-1 Gag-Pol polyprotein analysis. On panel **C**, the structure-structure LoCoHD distance matrix is shown, while on panel **D**, the average primitive atomic LoCoHD scores are illustrated in increasing order, along with their statistics. Panel **E** is a LoCoHD-RMSD scatter plot between the different elements of the ensemble, showing no correlation between these structural distances. Source data are provided as a Source Data file.

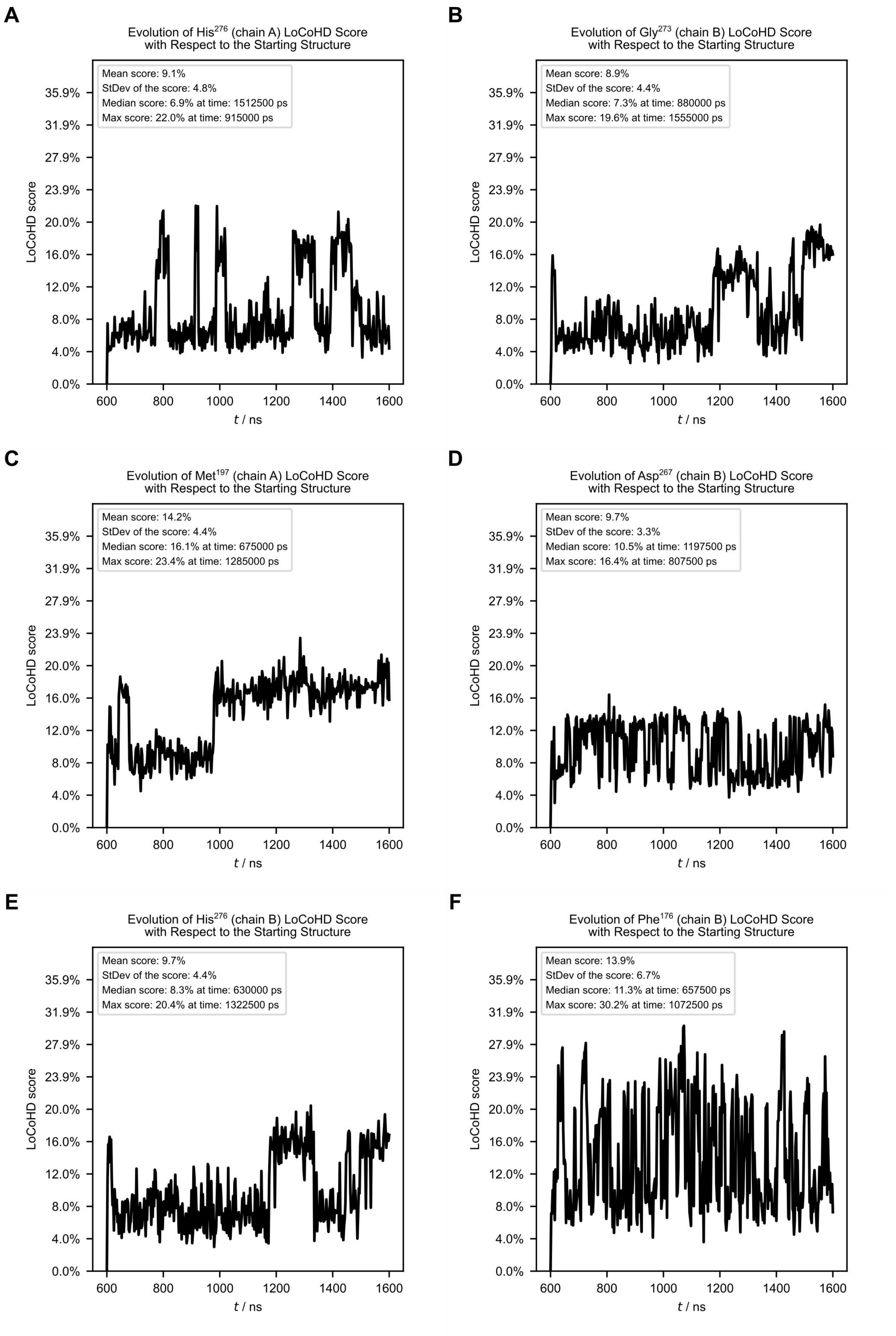

Supplementary Figure 18 depicting the time dependency of the LoCoHD scores of different residues in the podocin dimer MD simulation. (**Continued on next page**)

Supplementary Figure 18 (Continuation) The panels show the time dependency of residues with the highest Sarle's bimodality coefficients, indicating bimodality of the corresponding LoCoHD score distributions. Indeed, visual inspection of these plots reveals the fluctuation of these values between two extremities. The depicted residues are His^{276} (chain A), Gly^{273} (chain B), Met^{197} (chain A), Asp^{267} (chain B), His^{276} (chain B), and Phe^{176} (chain B) on panels **A**, **B**, **C**, **D**, **E** and **F**, respectively. Source data are provided as a Source Data file.

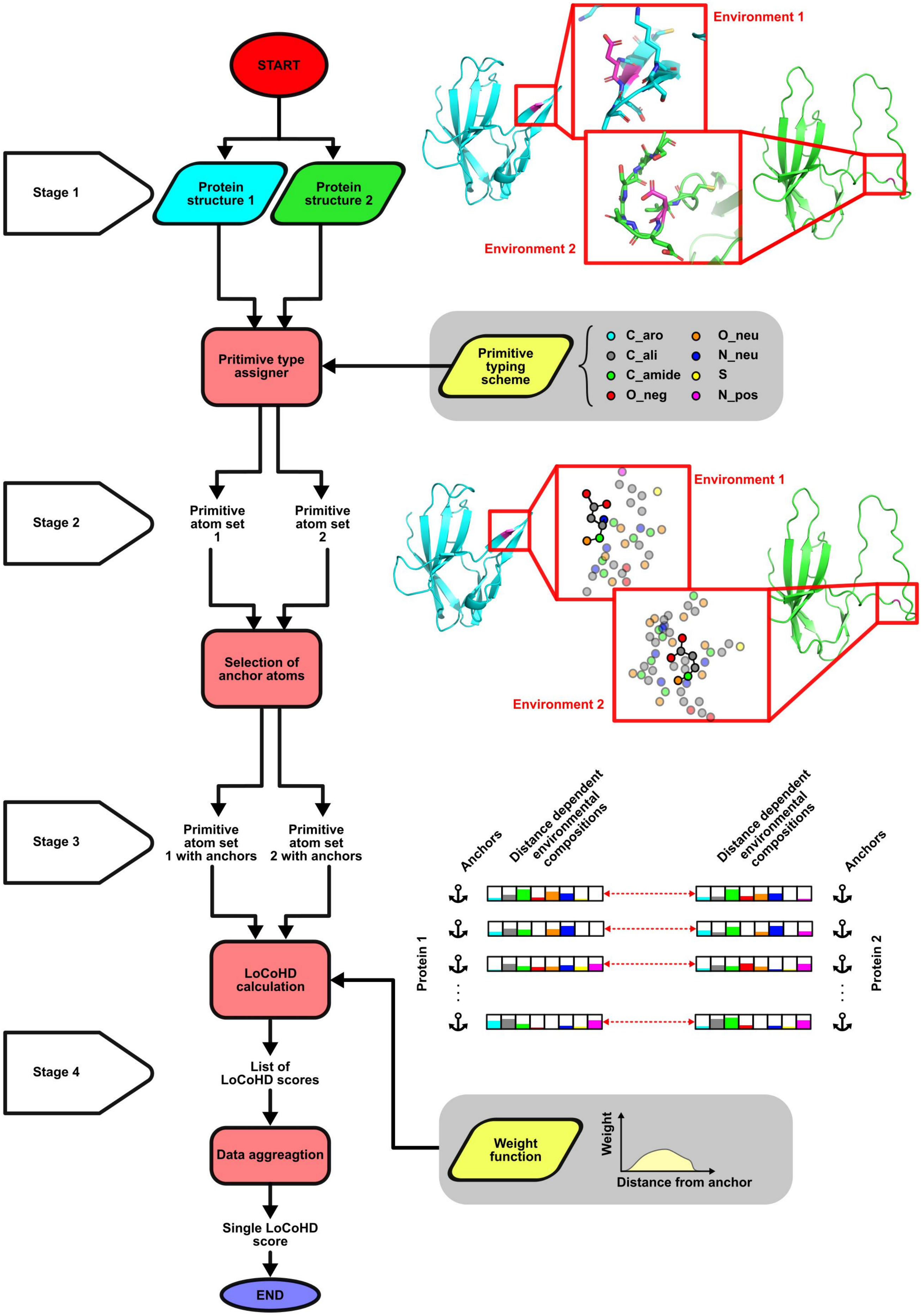

Supplementary Figure 19 An expanded flowchart for the LoCoHD algorithm. The algorithm first converts the real structures (Stage 1) to primitive structures (Stage 2), which are comprised of primitive atoms. Then, anchor atoms are selected from the primitive atoms (Stage 3) through of which the LoCoHD comparisons are performed (Stage 4). The resulting per-anchor LoCoHD scores can then be aggregated (e.g.: average or median calculation), which results in a single metric describing the difference between the structures.

Supplementary Note 6: Implementation Details

The LoCoHD algorithm is implemented in the Rust programming language for fast computation and is intended to be used as a Rust API for Python via the PyO3[25] crate. Primitive typing of protein structures is achieved in Python using the BioPython[26] package. Primitive typing schemes are saved as JSON files for easy and modular usage. Weight functions are implemented to contain the cumulative density functions (CDFs) of the following distributions; hyper-exponential distribution, Dagum distribution, uniform distribution, and Kumaraswamy distribution. Complete documentation can be found at **https://github.com/fazekaszs/loco_hd**.

Supplementary References

1. Kabsch, W. A solution for the best rotation to relate two sets of vectors. *Acta Cryst A* **32**, 922–923 (1976).

2. Kaindl, K. & Steipe, B. Metric properties of the root-mean-square deviation of vector sets. *Acta Crystallogr A Found Crystallogr* **53**, 809–809 (1997).

3. Steipe, B. A revised proof of the metric properties of optimally superimposed vector sets. *Acta Crystallogr A Found Crystallogr* **58**, 506–506 (2002).

4. Zhang, Y. & Skolnick, J. Scoring function for automated assessment of protein structure template quality. *Proteins* **57**, 702–710 (2004).

5. Holm, L. & Sander, C. Protein Structure Comparison by Alignment of Distance Matrices. *Journal of Molecular Biology* **233**, 123–138 (1993).

6. Wohlers, I., Domingues, F. S. & Klau, G. W. Towards optimal alignment of protein structure distance matrices. *Bioinformatics* **26**, 2273–2280 (2010).

7. Zemla, A. LGA: a method for finding 3D similarities in protein structures. *Nucleic Acids Research* **31**, 3370–3374 (2003).

8. Siew, N., Elofsson, A., Rychlewski, L. & Fischer, D. MaxSub: an automated measure for the assessment of protein structure prediction quality. *Bioinformatics* **16**, 776–785 (2000).

9. Ortiz, A. R., Strauss, C. E. M. & Olmea, O. MAMMOTH (Matching molecular models obtained from theory): An automated method for model comparison. *Protein Science* **11**, 2606–2621 (2009).

10. Mariani, V., Biasini, M., Barbato, A. & Schwede, T. lDDT: a local superposition-free score for comparing protein structures and models using distance difference tests. *Bioinformatics* **29**, 2722–2728 (2013).

11. Levitt, M. & Gerstein, M. A unified statistical framework for sequence comparison and structure comparison. *Proc. Natl. Acad. Sci. U.S.A.* **95**, 5913–5920 (1998).

12. Needleman, S. B. & Wunsch, C. D. A general method applicable to the search for similarities in the amino acid sequence of two proteins. *Journal of Molecular Biology* **48**, 443–453 (1970).

13. Olechnovič, K., Kulberkytė, E. & Venclovas, Č. CAD-score: A new contact area difference-based function for evaluation of protein structural models. *Proteins Struct. Funct. Bioinforma.* **81**, 149–162 (2013).

14. Krasnogor, N. & Pelta, D. A. Measuring the similarity of protein structures by means of the universal similarity metric. *Bioinformatics* **20**, 1015–1021 (2004).

15. Zhou, X., Chou, J. & Wong, S. T. Protein structure similarity from principle component correlation analysis. BMC *Bioinformatics* **7**, 40 (2006).

16. Galgonek, J., Hoksza, D. & Skopal, T. SProt: sphere-based protein structure similarity algorithm. *Proteome Sci.* **9**, S20 (2011).

17. Simonovsky, M. & Meyers, J. DeeplyTough: Learning Structural Comparison of Protein Binding Sites. *J. Chem. Inf. Model.* **60**, 2356–2366 (2020).

18. Hamamsy, T. et al. Protein remote homology detection and structural alignment using deep learning. *Nat. Biotechnol.* (2023) doi:10.1038/s41587-023-01917-2.

19. Chen, C., Chen, X., Morehead, A., Wu, T. & Cheng, J. 3D-equivariant graph neural networks for protein model quality assessment. *Bioinformatics* **39**, btad030 (2023).

20. Earnest, T., Fauman, E., Craik, C. S. & Stroud, R. 1.59 Å structure of trypsin at 120 K: Comparison of low temperature and room temperature structures. *Proteins Struct. Funct. Bioinforma.* **10**, 171–187 (1991).

21. Sprang, S. et al. The Three-Dimensional Structure of Asn 102 Mutant of Trypsin: Role of Asp^{102} in Serine Protease Catalysis. *Science* **237**, 905–909 (1987).

Supplementary References

22. McGrath, M. E., Haymore, B. L., Summers, N. L., Craik, C. S. & Fletterick, R. J. Structure of an engineered, metal-actuated switch in trypsin. *Biochemistry* **32**, 1914–1919 (1993).

23. Harmat, V. et al. Structure and Catalysis of Acylaminoacyl Peptidase. *J. Biol. Chem.* **286**, 1987–1998 (2011).

24. Song, J. et al. Intrinsically disordered γ-subunit of cGMP phosphodiesterase encodes functionally relevant transient secondary and tertiary structure. *Proc. Natl. Acad. Sci.* **105**, 1505–1510 (2008).

25. The PyO3 user guide. https://pyo3.rs (accessed: 2023-07-14).

26. Biopython. https://biopython.org/ (accessed: 2023-07-14).
